# Perspectives on high-quality interpersonal care among people obtaining abortions in Argentina

**DOI:** 10.1186/s12978-022-01401-1

**Published:** 2022-05-02

**Authors:** Chiara Bercu, Sofía Filippa, Ana Maria Ramirez, Anna Katz, Belén Grosso, Ruth Zurbriggen, Sandra Vázquez, Sarah E. Baum

**Affiliations:** 1 Ibis Reproductive Health, Oakland, CA USA; 2 Colectiva Feminista La Revuelta, Neuquén - Patagonia, Argentina; 3 FUSA para la salud integral con perspectiva de género y derechos, Asociación Civil, Buenos Aires, Argentina

**Keywords:** Abortion, Accompaniment, Reproductive health, Person-centred care, Quality of care, Argentina

## Abstract

**Introduction:**

Little is known about how people who have abortions describe high-quality interpersonal care in Argentina. This qualitative study aimed to understand preferences and priorities in their interactions with providers.

**Study design:**

We conducted 24 in-depth interviews with people who obtained abortions at a comprehensive reproductive health clinic or with support from a feminist accompaniment group in Buenos Aires and Neuquén, Argentina. We iteratively coded transcripts using a thematic analysis approach based on interpersonal domains present in current quality of care frameworks.

**Results:**

Participants described high-quality abortion care as feeling *acompañamiento* and *contención* from their providers – terms that imply receiving kind, caring, compassionate and emotionally supportive care throughout their abortion. They described four key elements of interpersonal interactions: attentive communication from providers and accompaniers, clear and understandable information provision, non-judgmental support, and individualized options for pain management.

**Conclusions:**

People obtaining abortions in Argentina consistently identified receiving compassionate and supportive care throughout an abortion as a key aspect of care. The findings have implications for incorporating people’s perspectives in the development of care guidelines, training of providers, and monitoring and improving of services. This is particularly important as the government of Argentina prepares to expand legal access to abortion.

**Supplementary Information:**

The online version contains supplementary material available at 10.1186/s12978-022-01401-1.

## Plain language summary

Little is known about how people who have abortions perceive the quality of the care they receive and what aspects of interpersonal interactions with providers matter to them. This qualitative study aimed to understand preferences of  people who had abortions at both a reproductive health clinic and with an accompaniment group in Argentina. We interviewed 24 people who obtained abortion care in Buenos Aires and Neuquén, Argentina. We asked them about their preferences for and experiences of abortion care. Then we analyzed the interviews, specifically assessing aspects of interpersonal care based on quality of care frameworks from the literature. Participants described high-quality abortion care as feeling *acompañamiento* and *contención* from their providers—terms that imply receiving kind, caring, compassionate and emotionally supportive care throughout their abortions. They described four key elements of interpersonal interactions: attentive communication from providers and accompaniers, clear and understandable information provision, non-judgmental support, and individualized options for pain management. In conclusion, people obtaining abortions in two distinct models of care in Argentina consistently identified receiving compassionate and supportive care throughout an abortion as a key aspect of care. The findings have implications for incorporating people’s perspectives in the development of care guidelines, training of providers, and monitoring and improving of services. This is particularly important as the government of Argentina prepares to expand legal access to abortion.

**Supplementary Information:**

The online version contains supplementary material available at 10.1186/s12978-022-01401-1.

## Background

In Argentina, abortion was legally restricted and regulated by the Penal Code up until January 2021. In January 2021, a law went into effect legalizing abortion up to 14 weeks gestation with no clausal restrictions [1]. Prior to the change in law, a legal abortion was only available if the pregnancy put the pregnant person’s^1^ life and/or health at risk, or if the pregnancy was a product of rape. While some provinces in the country gauranteed the legal termination of pregnancy when the person’s physical, emotional, or social wellbeing would be harmed by carrying the pregnancy to term, many provinces did not. Despite there being some legal access to abortion, numerous barriers prevented people from accessing care including lack of knowledge of the law, providers who refused to provide services, and stigma and normative beliefs around abortion [2, 3]. Abortion services were provided through a range of models of care in Argentina including comprehensive reproductive health clinics and hospitals which provided legal abortion services, and accompaniment groups which offered in-person and virtual information and support for people across the country who were self-managing medication abortions [4, 5]. Despite the legal and social barriers that existed to accessing care prior to January 2021, abortion in Argentina was common and people had safe and effective abortions across models of care. However, little is known about how people perceived the quality of their abortion services and what mattered to them in their interactions with providers.

Existing research on abortion quality of care has highlighted the importance of interpersonal care; interpersonal care refers to people's experiences and perceptions of the healthcare interaction [6]. A systematic review of published indicators for measuring quality of abortion services found that measures largely focused on structural and process aspects, such as infrastructure and technical competence, and only one third of the indicators were developed by incorporating abortion clients’ preferences [7]. Indicators to measure interpersonal care have not been consistently included; as such, there are gaps in our understanding of what aspects of the client-provider interaction matter most to people [8, 9]. The Interpersonal Quality in Abortion Care scale was developed in 2019 and sought to fill this gap by creating a measure derived from patients’ perspectives in the United States; the scale includes items related to respect, feeling listened to, and kindness [8]. These domains highlight the importance of interpersonal interactions in person-centered care.

Person-centered care is defined as an approach to care that is oriented around the patient’s identified needs, expectations and preferences [10]. At the root of person-centered care lie the interpersonal interactions between people and their providers, and providers’ ability to center the patient and their needs during care. Studies that address person-centered care in contraceptive and maternal health services have shown that women value interpersonal factors such as trust, empathy, respect, support, confidentiality and non-judgmental care from providers [11–14] and that these shape perceptions of quality and future care seeking behavior [15–17]. There is more recent evidence emerging within the context of abortion demonstrating how people's experiences of quality are positively impacted by person-centeredness [18], such as receiving comprehensive counseling, non-judgmental and supportive care, as well as feelings of autonomy and choice [18–20]. Though limited in number, studies from Latin America focusing on patient perspectives of abortion quality have found that client-provider interactions and information provision were identified as some of the most salient aspects of patients’ experiences [21, 22]. One study on quality of post-abortion care in public hospitals in Argentina found that, generally, care was not provided in a respectful manner and that key aspects, such as information provision, were not present [22].

Despite what we know about the importance of interpersonal interactions in quality of care, there are gaps in understanding preferences and priorities among people seeking abortion in different contexts and models of care, particularly outside of the healthcare systems. We analyzed interviews with people who had an abortion at either a comprehensive reproductive health clinic or with support from a feminist  accompaniment group and aimed to gain a deeper understanding of how people obtaining abortions experienced interactions with providers and what aspects of interpersonal care they valued. This analysis is particularly timely given the change in the law permitting abortion up to 14 weeks; we hope these qualitative data can inform integration of person-centered care in the implementation of legal abortion care throughout the country.

## Methods

The interviews analyzed for this manuscript were part of a larger study conducted in four countries (Argentina, Bangladesh, Ethiopia and Nigeria), which aimed to gain a deeper understanding of people’s^2^ experiences with abortion services and their perceptions of quality of care [23]. For this manuscript, we analyzed semi-structured in-depth interviews with people who obtained an abortion in Buenos Aires and Neuquén, Argentina.

Participants were recruited from two different abortion care organizations—through a feminist accompaniment group based in Neuquén and a comprehensive reproductive health clinic in Buenos Aires. The feminist accompaniment group , Colectiva Feminista, La Revuelta (La Revuelta), hosts group information sessions where people seeking an abortion learn how to have a safe self-managed abortion and complete a medication abortion at home. La Revuelta is made up of trained volunteers, or ‘accompaniers,’ who provide evidence-based support and information by telephone and in-person throughout the medication abortion process to individuals seeking an abortion. In this paper, the term “accompaniers” is used to refer to members of the feminist collective, La Revuelta, who provide abortion information and support. The reproductive health clinic, Casa FUSA, Centro para Atención de Adolescentes y Jóvenes (FUSA), is a private care facility that provides in-clinic abortion care in Buenos Aires. Although FUSA also offers medication abortion, for this investigation we only recruited individuals who had received a manual vacuum aspiration (MVA), a common procedure for induced abortion or post-abortion care [24]. In our study, the people who had their abortions with FUSA visited the clinic prior to their abortions to participate in both a group information session and to have an individual counseling session with a provider and then returned a few days later for their MVA. During the group information session, people were given generalized information about abortion, the legal status of abortion, abortive methods and safety and efficacy; while the individual counseling session was a space for participants to select their abortive method and speak with providers about any individual concerns they did not feel comfortable raising in a group setting. In this paper, the term “providers” is used to refer to both medical personnel and staff who provide abortion care at FUSA. Given that recruitment sites for this study were run by feminist activists and a non-profit organizations, it is likely that the services they provided were of higher quality than average public and private sector facilities in Argentina.

We conducted 24 semi-structured in-depth interviews, 12 at each recruitment site, from November 2018 to January 2019. The sample size was determined as part of the larger study. We sought to conduct approximately 100 interviews, split equally among the four countries. In Argentina, we decided to divide the interviews equally among both models of care. Trained staff at each site recruited participants either prior to their abortion during a group information session or after their abortion was complete by telephone. All interviews were conducted after the abortion was complete. To be eligible for the study, participants had to be aged 15 years or older, able to provide informed consent, able to speak Spanish, and have had an abortion with La Revuelta or FUSA within the 6-months prior to being recruited into the study.

One interviewer (BG) conducted all of the interviews in person in Neuquén and Buenos Aires. BG identifies as a woman and had extensive academic training and experience in conducting ethical research, qualitative methods, and qualitative interviewing techniques. All interviews were conducted at a space provided by La Revuelta or FUSA or in a secure location of the participant’s choosing. In order to maintain participant privacy, given the sensitivity of the subject matter, BG and the participant were the only people present during interviews. After each interview, BG made field notes. BG was associated with La Revuelta at the time of the study, but did not serve as an accompanier for, and had not previously met, any of the people recruited to participate.

The interview guide was developed for the larger study based on quality of care frameworks [9, 25, 26] as well as previous studies on abortion quality. The guide was translated to Spanish, piloted, and adapted to the Argentine context to ensure clarity or cultural applicability. The interview guide included questions on participant’s abortion experiences as well as interpersonal interactions with providers (See Additional file 1—Interview Guide in Spanish). The interviews lasted between 40 and 60 min and were audio-recorded. Participants were compensated with a public-transportation card with a value of approximately $10 USD. This study was approved by Allendale Investigational Review Board based in the United States and Comité de Bioética de la Fundación Huésped, based in Argentina.

We conducted thematic analysis in order to explore people's perceptions and experiences of interpersonal care during their abortions. All interviews were professionally transcribed and analyzed thematically in Spanish. We developed an initial codebook using key themes from the interview guide, domains of quality of care drawn from a literature review on the subject, and emergent codes from the transcripts. Two members of the research team (CB and SF) double coded two transcripts, after which they met to discuss discrepancies in coding, adjusted, redefined or modified codes and code descriptions and then applied the final codebook to all 24 transcripts. We coded using MAXQDA 2018 (VERBI Software). We analyzed emergent themes during the process of writing code summaries and assessed patterns as well as outlying trends within the data set. Our sample size was not sufficient to conduct comparative analyses between the two models of care, therefore we analyzed the range of experiences across the entire dataset. When differences in care provision were noted based on the model of care, we include these distinctions in the results. Key staff at recruitment sites contributed to the interpretation of data by reviewing preliminary findings. Quotes have been translated into English for publication. Original quotes, in Spanish, are available in Additional file 2–Quotes in Spanish and English.

## Results

Amongst the 24 participants, the average age was 30 years old, ranging between 20 and 41 years old. A quarter of participants reported having an abortion after 12 weeks’ gestation. Half of all participants had a medication abortion (all recruited from the feminist accompaniment group) and half had a MVA (all recruited from the clinic). There were six participants who reported having a prior abortion and nine who already had children. None of the participants reported being married, however nearly half of participants reported being in a relationship, four of whom lived with their partners. Ten participants reported having a paid job while five reported being full-time students; six participants were both students and had a paid job and three participants reported being unemployed at the time of the interview. (Table 1).

**Table 1:** Participant characteristics

	**N = 24** **n (%)**
Age (years)	
Mean	30
Range	20–41
Abortion method	
Medication abortion	12 (50.0%)
Manual vacuum aspiration (MVA)	12 (50.0%)
Gestational age	
< = 12 weeks	16 (66.6%)
> 12 weeks	6 (25.0%)
No data	2 (8.3%)
Previous abortion	
Yes	6 (25.0%)
No	18 (75.0%)
Children	
Yes	9 (37.5%)
No	15 (62.5%)
Marital status	
Married	0 (0.0%)
In a relationship	11 (45.8%)
Single	13 (54.2%)
Employment status	
Employed	10 (41.7%)
Student	5 (20.8%)
Employed & Student	6 (25.0%)
Unemployed	3 (12.5%)

### Acompañamiento and Contención^3^

Universally, participants recruited from both the clinic and accompaniment models of care described high-quality abortion care as feeling *acompañamiento* and *contención* from their providers. These terms emerged over and over in the descriptions of positive interactions, and were highlighted across the sample as necessary for good care. Interpersonal care provided with *acompañamiento* and *contención* helped participants trust their provider or accompanier, which, in turn, reassured their sense of safety throughout the abortion. When describing their experience with the accompaniment group, a participant explained*, “I always say that they provide you with a security that, out there, perhaps at another place, I do not know if…let’s see, they are not doctors, but the fact that they provide such contención is really important, you do not feel alone” (Age 34, Accompaniment)*. Another participant, who did not experience any abortion symptoms after three medication abortion attempts, echoed the importance of knowing that they would not be left alone. With the support of their accompanier, the participant ultimately ended up receiving an MVA at a clinic,

*“What I always felt was that sense of security, you are not going to come out of this alone, we are never going to abandon you, this is where we solve this. That kind of...gives you that sense of security, to say I failed once and now what do I do, maybe they will leave me on my own, no, they will not leave you alone, because they told me, ‘we already started to dance, we are going to continue dancing until the song is over’, and that is how it is.” (Age 33, Accompaniment)*

*Acompañamiento* and *contención* also played a role in relieving participants’ anxieties. Participants described a sense of respite that was rooted in the realization that they would be able to end their pregnancies in a context of high-quality care. A participant described, *“I felt…how can I say it, very well attended to, I did not expect to get here, very emotionally contenida.” (Age 26, Clinic)*. Finally, a participant expressed that they felt “light” after meeting with the accompaniment group, since they knew that the accompaniers would help them terminate their pregnancy, *“I felt…I felt light, I felt supported, acompañada, and I knew that everything would be fine, so I felt calmer, much calmer, because I was very scared, I felt that my problem was solved, that is what I felt” (Age 26, Accompaniment).*

Below we present four key aspects of abortion care with *acompañamiento* and *contención* which help to define what participants in Argentina valued during interpersonal interactions: attentive communication from providers and accompaniers, clear and understandable information provision, non-judgmental support, and individualized options for pain management.

### Attentive communication and interactions

Many participants in the study described feeling supported during interpersonal interactions when providers and accompaniers communicated continuously and were attentive to their needs throughout their abortion process, particularly during the abortion procedure itself. One participant at the clinic appreciated the fact that the provider explained every step of their MVA as it was taking place, and showed attentiveness to their comfort,

*“Well, I can’t remember the name of the girl who was accompanying me, but well, she was sitting next to me, talking to me, as if we knew each other forever, the doctor too, who was there, was telling me everything she was doing. That everything was going well. Always asking me if it hurt, what I felt, if I felt something strange.” (Age 25, Clinic)*

When providers and accompaniers showed concern about their physical and emotional wellbeing, participants felt safer, calmer and more trusting of the provider or accompanier. One participant highlighted the fact that their provider asked for permission to touch them before beginning the MVA procedure, which they did not anticipate. This helped them feel that they were receiving respectful care,

*“They were very attentive because they told me, ‘I’m going to put this on you, I’m going to put that on you, I’ll touch you here.’ So these are things that you are not accustomed to happening either. No specialist is going to ask you for your permission.” (Age 41, Clinic).*

Participants accompanied by the feminist accompaniment group described feeling *contención* and *acompañamiento* while interacting with their accompanier from the beginning to the end of their experience. This participant explained the importance of knowing the accompanier would be there for them if something happened,

*“From the day you are going to do it, they are already accompanying you, that is, it is not that they are just going to show you how to do it…there is a before, during and after, and everything is with acompañamiento, and that is really good, because you know that in the moment if something happens to you it is not that you go to a hospital or something, you have someone to consult, who can help you.” (Age 30, Accompaniment)*

In some cases, participants were surprised by how supported they felt by the interactions they had with accompaniers over phone calls and text, such as this participant,

*“But it was like… it was very relieving and I felt the contención beyond the kilometers and beyond the non-contact face to face, I felt the contención and I knew they were there, it was not the only time they called me… they explained everything to me, they called me before, after the process, and during the process too.” (Age 30, Accompaniment)*

The attentiveness that both accompaniers and providers showed towards participants helped them feel that they could rely on the providers or accompanier, which helped participants feel that they were very well cared for and supported during their abortions.

### Comprehensive, clear information provision

Almost all the participants described the importance of receiving clear and comprehensive information from their providers or accompaniers. At both recruitment sites, comprehensive information, which included details on each step of the abortion process, was provided during an initial group information session, as this participant explains,

*“They explained everything step by step, and it was all like, ‘yes, let’s go, I want to end [my pregnancy] here’, and nothing, in terms of contención here I found it 100%, that is, contención that I did not find in him [my partner], that I did not find in my friend or in my psychologist.” (Age 20, Clinic)*

For the participant, the detailed information they received on the procedure helped them make the choice to have their abortion at the clinic. Additionally, this participant linked information provision to feeling *contención* at the clinic and having a sense of support that they had not felt or received from others. This highlights how providing transparency on the abortion procedure may have facilitated a sense of trust between the participants and the providers.

Participants also appreciated the opportunity to ask questions and discuss their circumstances with providers and accompaniers, as well as with other people who were there to have an abortion. One participant mentioned that they were given the opportunity to interact with the MVA instruments prior to the procedure in the clinical model. Additionally, the group information session that both models used offered a space for participants to hear what questions other people had and instilled in them a sense of community and safety. As one participant explained,

*“This thing of it being in a group makes you feel a little more relaxed, because you know that you are not alone and that there others are going to ask questions that do not occur to you and that could happen to you, so, then you are like more sure of what you are doing.” (Age 30, Accompaniment)*

Various participants also highlighted the importance of receiving information that was easily accessible and understandable, as this participant explains,

*“It was like they spoke to you in a way that you could understand everything, you understand? And I came without knowing anything...the truth is that they inspired so much confidence in me, so much security, they explained everything, absolutely everything.” (Age 32, Clinic)*

Receiving thorough information helped participants to feel safe, helped them trust their providers and helped them feel well-prepared for their abortion experiences.

### Empathetic and non-judgmental listening

Participants often mentioned the importance of feeling heard by their providers or accompaniers. This made them feel respected and understood by their providers and accompaniers. One participant explains*, “They provide contención emotionally, they listen to you, they know how to listen, they know how to understand the situation you are going through and professionally they are very delicate, respectful, attentive”* (Age 26, Clinic). Another participant described how it felt to be able to share their motives for wanting an abortion with a provider who helped them feel that their experience was important,

*“I know that a lot of girls pass through here, but they like made you feel, I don't know, not special, but like it mattered what you were feeling, and that your decision was fine, whatever it was, that the reason why you didn’t want to have it [the pregnancy] mattered.” (Age 20, Clinic)*

Perceiving that providers and accompaniers did not cast judgement or stigmatize their decision, and instead listened empathetically, helped participants feel that they were receiving care with *acompañamiento* and *contención*.

Participants also recounted that in both models of care, they felt that providers and accompaniers took efforts to normalize abortion. One participant explains of the accompaniment model*, “since here [abortion] is normalized, what we do is not judged in any way” (Age 33, Accompaniment)*. The group information sessions were also instrumental in normalizing abortion. One participant who was initially skeptical about the group information session recounts how they later understood it as a way of legitimizing abortion,

*“Why do I have to go to a collective interview?’ At first I didn’t like that one bit and after, I understood why it was collective, or at least I explained it to myself through the experience we had, and I liked that...it’s good thinking from the perspective of taking it [abortion] out of the dark or the clandestine or illegal practice or secret practice, the fact that it is collective.”* (Age 36, Clinic)

Another participant accompanied by the hotline model further explained how the group information sessions helped to create a friendly rapport between participants and accompaniers, *“The girls made it more enjoyable, it seemed like a chat with friends. Yes, it was really good. Yes, I felt very good, I felt contenida, it was good” (Age 30, Accompaniment)*. The non-judgmental space that providers and accompaniers helped to facilitate during group information sessions, as well as the empathetic listening that participants recounted receiving from their providers and accompaniers, helped them feel heard, validated and supported and was an important aspect of receiving care with *acompañamiento* and *contención*.

### Choice in pain management

Participants reported feeling that providers and accompaniers took their needs into account and offered them both analgesic and innovative non-medical techniques for pain management. For instance, participants who received care at the clinic were given a number of options for pain management including hot water bottles to place on their abdomens and the opportunity to play the music of their choice during their MVA. One participant described how the music and the conversation with the people in the room helped distract them from the pain,

*“They put music on for me, Los Redondos [a band], and the truth is that if I’m honest with you, I didn’t feel any pain,…the girls talked to me and we chatted, and I had the hot water bottle, for like in case you feel pain.” (Age 32, Clinic)*

When reflecting in the interviews, participants often highlighted these moments as representative of supportive interpersonal interactions during their abortion care. Another participant who received care at the clinic described being surprised at how much effort their providers made to meet their individual comfort needs,

*“They told me something that really surprised me, they said ‘if you want, we can play music’, which surprised me in the best sense...at what level they are, I don’t know how to say it, as if they are really thinking about the comfort of the patient, in a moment like that, I had the best time possible.” (Age 21, Clinic)*

As this participant points out, the ability for many participants to have choice in their pain management helped them feel as though they had the best possible healthcare experience. In the feminist accompaniment group, accompaniers suggested that participants try different pain management options at home, for example using a hot compress, or setting up the space in which they were going to take the medications so that it would feel unique and comfortable. For instance, one participant mentioned watching their favorite TV show and preparing some of their favorite snacks, as their accompanier had suggested,

*“She [the accompanier] told me to stay calm, that the calmer I was, the faster and easier it would be, she told me to create a nice ambiance, that the person who is with you transmits good things to you, if you like incense, light an incense stick, prepare whatever you like to eat…I made myself a cake of dulce de leche [caramel], walnuts and chocolate…she told me watch whatever you want, so I did it watching Ru Paul...[television show].” (Age 30, Accompaniment)*

The empathic and compassionate way through which providers and accompaniers centered the needs and choices of participants in relation to pain management helped them feel as though their individual needs were being taken into account and helped them feel that they were in a safe and comfortable environment during their abortions.

## Discussion

The results of this qualitative study provide insights into the elements of interpersonal care that were most salient for people having abortions through clinical and accompaniment models of care in Argentina. The specific examples of positive interactions with providers and accompaniers help shed light on what participants looked for in a high-quality abortion experience. Participant’s highlighted open communication, comprehensive information provision, non-judgmental care and choice in pain management as key components of their abortion experiences. Study participants consistently reiterated how imperative it was and how much they appreciated interpersonal care that was provided with *acompañamiento* and *contención* – continual emotional support, empathy, and understanding.

These findings contribute to a growing body of literature that recognizes interpersonal care as a priority and document the value of person-centered, empathetic, and emotionally supportive interactions with providers. Our results echo a recent study with abortion clients in Kenya that highlighted the importance of receiving respectful, supportive care continuously throughout their abortion process [19]. Other studies have found that, while important, person-centered care is often absent from abortion services around the globe [27, 28]. The current findings encourage us to consider quality of care, and specifically interpersonal care, as necessary not only during a single encounter with the provider, but from the time a person decides to have an abortion until the process is complete. This might include various types of providers for an individual, such as conversations with a primary care physician or accompanier, front desk staff, an ultrasound technician, pharmacists selling abortion pills, or providers offering follow-up services. Our findings complement other studies that show that people appreciate compassionate and supportive communication that is provided not just in-person but also virtually by telephone, online platforms or texting systems [29, 30]. In legally restrictive settings where people seeking abortion may not have access to legal clinical services, and particularly during restrictions associated with the global pandemic, these alternative mechanisms for communication have become more relevant and therefore quality must be monitored.

This study highlights various innovative ways of providing person-centered interpersonal interactions. Participants from both the clinical model and the accompaniment model noted benefits of the pre-abortion group information sessions. While studies have shown that group counseling is not private enough for some [31], participants in this study nearly all felt the sessions were valuable even if they were hesitant at first; they listened to each other’s questions, felt less isolated, and the group atmosphere may have contributed to normalizing abortion experiences amongst participants. In addition to the group information sessions, La Revuelta and FUSA provided ample opportunities for participants to ask questions and received support from providers one-on-one. Some participants reported being impressed by the providers’ and accompaniers’ attempts to make the abortion experience not only comfortable, but even enjoyable. This included playing music during in-clinic abortions, offering hot water bottles during and after their procedures, and providing suggestions for how to set up their spaces at home to ease the self-managed abortion experience. These innovative approaches centered people’s needs and helped them feel cared for and supported during their abortion. Strategies that help to normalize abortion and offer both choice and autonomy to people seeking abortions are particularly noteworthy within abortion care as they help people feel empowered in their decisions and may help tackle some of the internalized stigma or social stigma surrounding abortion.

This study adds nuance to domains that have emerged in person-centered care frameworks in the literature. The details offered in these narratives have implications for how we develop or adapt tools to measure quality of care from the perspective of people who have abortions. For example, in addition to receiving comprehensive information about the abortion process, participants felt that it was important that the information be provided in a way that helped them feel heard, comforted, validated, and free to ask clarifying questions. Participants also noted their appreciation for having choice in pain management during their abortion both at the clinic during surgical abortion and at home for medication abortions. If indicators simply address whether a person received information or pain management, they may not successfully assess *how* they received that element of care. Conversely, if indicators simply ask if a person felt they received respectful compassionate care, they may not successfully capture at what point during the process the person did, or did not, feel respected. Sudhinaraset et al. recently developed and validated a person-centered abortion care scale based on data from abortion clients in Kenya that included sub-scales to measure respectful and supportive care as well as communication and autonomy [32]. These domains are reflected in our data, which suggests that key aspects of person-centered care may carry over across political and social contexts. For example, the detailed descriptions of high-quality care from this study in Argentina can contribute to future research exploring how this scale or future measures could be adapted for the Argentinian context. This is particularly important as the government prepares to expand legal abortion access in public facilities across the country. It will be critical to incorporate the values and preferences of people who obtain abortion in the development of care guidelines, training of providers, and monitoring and improving of services.

There are a number of limitations that should be considered when interpreting the findings in this study. First, this study recruited participants from two sites that were known to have high-quality abortion care and therefore do not represent experiences across a range of services. While further research is needed to understand the experiences of people in public facilities, the current findings offer unique insight into preferences and priorities in interpersonal care. Second, we did not ask people about their gender identity, as such, we cannot say anything about the range of genders present within this manuscript or how gender identity may have impacted people’s experiences of abortion care. Third, both recruitment sites were based in urban areas, therefore the perspectives are not representative of rural areas of Argentina. Lastly, providers and accompaniers at the two sites were responsible for inviting people to participate in the study, which may have influenced who decided to participate and could have limited the negative feedback shared in the interviews.

## Conclusion

This study identified elements of interpersonal care that were most salient to people seeking abortions at clinics and with accompaniment groups in Argentina. Our findings suggest that receiving compassionate and supportive care throughout an abortion was consistently a key aspect of care. While cultural and legal contexts may shift what is most important for people seeking abortions, we posit that the feeling of trust, empathy and respect from providers, which emerged from this study, is applicable across settings and models of care. Given the global shift towards medication abortion and the recent expansion of telemedicine during the Covid-19 global pandemic, this study reminds us that interpersonal interactions are highly important for people’s overall experience of quality of care even when abortion support is being provided virtually. Furthermore, given this critical moment in abortion provision in Argentina, we hope that these findings will inform the provision and evaluation of person-centered abortion care in the country.

## Supplementary Information

**Additional file 1.** Interview Guide in Spanish.

**Additional file 2.** Quotes in Spanish and English.

**Additional file 3.** Spanish version of article.

## Data Availability

The dataset generated and analyzed during the current study is not publicly available in order to maintain confidentiality and reduce risk to the participants. The interview guide is published as an appendix.

## Abbreviations

Casa FUSA: Centro para Atención de Adolescentes y Jóvenes (FUSA): A comprehensive reproductive health clinic in Buenos Aires
La Revuelta: Collectiva Feminista La Revuelta
MVA: Manual Vacuum Aspiration

## Footnotes

1 We use ‘pregnant person’ and ‘people’ in this paper whenever possible to acknowledge the diversity of people with uteri and the diversity of pregnancy and abortion experiences. We specify ‘women’ when people are referred to as women by authors of other publications. We recognize that the experiences of intersex, trans and non-binary individuals are underrepresented in the literature on pregnancy and abortion and that this analysis was not structured to address that gap.

2 In this manuscript we use the term ‘people’ to describe our study population since we did not ask participants about their gender identities.

3 These are two key terms in Spanish that participants used frequently to describe the way that providers/accompaniers interacted with them. In an effort to capture the essence of these words, we will continue to refer to the two terms in Spanish throughout this paper. “*Contención*”, and its adjective “*contenida*” literally translate to “containment” in English. However, in use, the word suggests a type of kind, caring, compassionate emotional support, a support that contains the individuals feeling of fear or anxiety. “*Acompañamiento*”, and its adjective “*acompañada*” literally translate to “accompaniment” in English. But in practice, it too suggests kind, caring, and supportive care that accompanies one throughout their healthcare experience.
