# Supplementary material for: Perspectives on high-quality interpersonal care among people obtaining abortions in Argentina
Source: Reprod Health. 2022 May 2;19:107. doi: 10.1186/s12978-022-01401-1 (PMC9059438; doi:10.1186/s12978-022-01401-1)
Supplement: Supplementary file 1 — Additional file 1. Interview Guide in Spanish. [file 12978_2022_1401_MOESM1_ESM.docx]

***[Nota a entrevistadora: las instrucciones van a estar en negrita para que sean fáciles de leer; no debieran ser dichas en voz alta]***

Hola, mi nombre es ____________. Estoy conduciendo esta entrevista de parte de ***[organización]*** e Ibis Reproductive Health. Muchas gracias por acceder a participar. Quiero resaltar que para asegurarse de que las mujeres reciban la mejor calidad de servicios, te estamos pidiendo que compartas tus pensamientos honestos sobre los servicios que recibiste. Nada de lo que digas hoy va a lastimar mis sentimientos, y nada de lo que digas te va a ser atribuido, y no va a afectar tu habilidad para acceder a servicios en el futuro.

Como mencioné en el proceso de consentimiento, hoy me gustaría explorar tus pensamientos, opiniones, y experiencias con servicios de salud sexual y reproductiva, incluyendo aborto. Lo que compartas nos ayudará a mejorar la calidad de estos servicios. No estoy buscando un tipo de respuesta en particular, solo tu opinión honesta.

Para proteger tu confidencialidad y privacidad, tu nombre nunca será usado en conexión a la información que compartas. Por favor recordá que tu participación es puramente voluntaria, no necesitás responder ninguna pregunta que te incomode, y podés terminar la entrevista en cualquier momento sin ningún tipo de penalización.

Vamos a empezar. Voy a empezar a grabar ahora.

***[Empezar grabación]***

***[Decir el número de identificación, nombre de la entrevistadora y la fecha al comienzo de la grabación]***

**Sección 1. Introducción**

Me gustaría empezar preguntándote un poquito sobre vos.

1. ¿Me podrías contar un poco sobre vos? *[Por ejemplo edad, trabajo, familia, etc.]*

**Sección 2. Definiendo cuidado de alta calidad**

Ahora voy a hacer una transición para preguntarte sobre servicios de salud. Por favor pensá sobre cuando vos o tu familia han estado enfermos y necesitaron buscar cuidado médico.

1. ¿En dónde buscan servicios de cuidado médico vos o tu familia usualmente?
   1. ¿Por qué van a buscar servicios de cuidado médico allá?

A veces recibimos servicios de salud que son buenos, a veces muy malos, y a veces en el medio.

1. Por favor describí un momento reciente en el que hayas recibido cuidados de salud muy buenos de un doctor/a, enfermero/a o algún otro proveedor de servicios o cuidados de salud.
2. ¿Qué hizo que el servicio sea muy bueno?
3. Ahora por favor describí un momento reciente en el que vos o tu familia hayan recibido cuidados de salud inaceptables o malos de un proveedor de salud.
   1. ¿Qué hizo que el cuidado sea malo?
4. En tu opinión, ¿qué es lo que hace que algunos servicios de salud sean buenos o sean malos?
5. ¿A dónde van las mujeres en tu comunidad para obtener cuidado prenatal?
   1. ¿Y para métodos anticonceptivos?
   2. ¿Cómo saben las mujeres en tu comunidad a dónde ir para obtener buenos cuidados para estos servicios?

**Sección 3. Antes del aborto**

Ahora me gustaría preguntarte algunas preguntas sobre tu embarazo reciente.

1. ¿En general, cómo te sentías o cuáles eran tus creencias respecto al aborto en general antes de decidir tener un aborto?
2. ¿Qué habías escuchado sobre el aborto antes de obtener servicios de aborto?
   1. ¿Sobre la seguridad o riesgos potenciales del aborto?
   2. ¿Sobre los riesgos o consecuencias potenciales después de un aborto?
   3. ¿Sobre cómo serías tratada?
   4. ¿Sobre las leyes sobre cuando una mujer puede obtener un aborto?
3. ¿Dónde aprendiste esta información?
4. ¿Cómo decidiste buscar servicios de aborto?
5. ¿Hiciste algo para intentar terminar con ese embarazo antes de contactar a La Revuelta/FUSA? Contame sobre eso.
6. ¿Cómo te enteraste de La Revuelta/FUSA?
   1. ¿Qué habías escuchado sobre La Revuelta/FUSA?
7. ¿Qué te hizo decidir buscar servicios de La Revuelta/FUSA?
   1. ¿Escuchaste historias sobre otras mujeres obteniendo servicios de aborto que influenciaron tu decisión sobre dónde ir? Contame sobre eso.
8. ¿Qué expectativas tenías sobre cómo serías tratada al recibir cuidados de aborto?
   1. ¿Y tenías algún miedo? ¿Cuál era tu mayor miedo?
9. ¿Hablaste con un/a proveedor/a de salud sobre tu embarazo antes de contactar a La Revuelta/FUSA por primera vez?

***[SI ES ASI]***

1. Contame sobre eso.
2. ¿Qué te dijeron?
3. ¿Cómo te trataron?
4. ¿Más o menos cual era tu edad gestacional cuando contactaste a La Revuelta/FUSA? Sentite libre de hacer una estimación si no te acordás exactamente.

**Sección 4. Durante el aborto**

Tengo curiosidad de escuchar más sobre tu experiencia con la telefonista/acompañante

1. ¿Cómo fuiste tratada durante tus interacciones con La Revuelta/FUSA?
   1. ¿Qué te hizo sentir de esa manera?
2. ¿Hay algo que dijeron que te asustó? ¿Por qué?
3. ¿De qué formas te hicieron sentir preparada o no preparada para el aborto?
4. ¿Qué tipo de información recibiste de parte de la telefonista/acompañante que haya sido difícil de entender, si es que esto ocurrió?
5. ¿Qué hizo la telefonista/acompañante para proteger o no tu privacidad?
   1. ¿Qué tan importante fue esto para vos?
6. ¿En algún punto te sentiste juzgada durante tu experiencia con La Revuelta/FUSA? Contame sobre eso
7. ¿Tuviste alguna otra interacción negativa con otras telefonistas/acompañantes? Contame sobre eso.
8. Contame sobre la información que recibiste sobre anticonceptivos que recibiste después de tu aborto.
   1. ¿Había alguna información que querías pero que no recibiste en relación a métodos anticonceptivos? Contame sobre eso.
9. ¿Quién estaba con vos cuando tomaste las pastillas, si es que había alguien? ¿De qué formas te apoyó o no esta persona?
10. ¿Cuántas veces hablaste con la telefonista/acompañante durante todo el proceso?

**Sección 5. Después del aborto**

1. ¿Fuiste a ver a un doctor/a o fuiste a una clínica después del aborto/terminar el embarazo? ¿Por qué si? ¿Por qué no?

***[SI ES ASI]***

1. ¿Qué le dijiste al/a la proveedor/a sobre lo que pasó?
2. ¿Qué tipo de tratamiento médico recibiste?
3. ¿Cómo te trataron?
4. ¿Cuál fue la mejor parte del cuidado que recibiste? ¿Por qué?
5. ¿Cuál fue la peor parte del cuidado que recibiste? ¿Por qué?
6. Pensando sobre el costo del aborto, ¿cómo lo describirías? ¿Qué tan manejable fue para vos?
7. ¿Creés que tu edad afectó la manera que fuiste tratada?
   1. ¿Creés que la experiencia hubiese sido igual o distinta para una amiga tuya que es mucho más grande que vos? ¿Más chica? ¿Por qué?
8. ¿Creés que tu estado civil afectó cómo fuiste tratada?
9. ¿Creés que la experiencia hubiese sido igual o distinta para una amiga tuya que está **(*casada or no casada)***  ***[Elegir la experiencia opuesta de la participante]***

**Sección 6. Reflexiones sobre la experiencia del aborto**

1. Si alguien nunca hubiese escuchado de La Revuelta/FUSA, ¿cómo las describirías a esta persona?
2. ¿Qué hubiese hecho la experiencia con La Revuelta/FUSA mejor?
3. ¿Cómo se compara, o no, tu experiencia recibiendo servicios de aborto con respecto a tus expectativas?
4. ¿Habías tenido una experiencia de aborto antes de este embarazo reciente?
5. ¿Y cómo se compara esta experiencia con tu aborto previo?

***[Por ejemplo: seguridad, dolor, prontitud y experiencia con proveedor/***

1. ¿Qué consejo le darías a una amiga que también está buscando cómo terminar su embarazo? ¿Qué debería saber?
2. Si tuvieras que describir las tres partes más importantes del mejor cuidado de aborto, ¿qué tres partes dirías? Por favor mencioná las partes que sientas que son las más importantes para vos, sin importar que tan grandes o chicas.

Por último…..

1. ¿Cómo fue participar en esta entrevista?
2. Antes de terminar, me gustaría preguntarte algunas preguntas más sobre vos. Le preguntamos esto a todas las personas con las que hablamos.
3. ¿Cuántos años tenés? _________
4. ¿Cuál es tu estado sentimental en este momento?
5. ¿Cuántos hijos tenés, si es que tenés hijos? ____________
6. ¿Estás trabajando en este momento? ¿Estudiando? ¿Las dos?

**-- Gracias por tomarte el tiempo de hacer esta entrevista con nosotras--**
