# Supplementary material for: Perspectives on high-quality interpersonal care among people obtaining abortions in Argentina
Source: Reprod Health. 2022 May 2;19:107. doi: 10.1186/s12978-022-01401-1 (PMC9059438; doi:10.1186/s12978-022-01401-1)
Supplement: Supplementary file 2 — Additional file 2. Quotes in Spanish and English. [file 12978_2022_1401_MOESM2_ESM.docx]

**Supplementary File II: Quotes in Spanish and English**

**Theme: *Acompañamiento* and *Contención***

| **Spanish** | **English** |
| --- | --- |
| “[Y]o siempre digo que te brindan una seguridad que por ahí capaz que en otro lugar no sé si… a ver, no son médicos, pero el hecho de la contención es re importante, no te sentís sola” (Age 34, Accompaniment). | “I always say that they provide you with a security that, out there, perhaps at another place, I do not know if...let’s see, they are not doctors, but the fact that they provide such *contención* is really important, you do not feel alone.” (Age 34, Accompaniment) |
| “Lo que sí siempre sentí como esa seguridad, de esta no vas a salir sola, no te vamos a abandonar jamás, de esta solucionamos esto. Eso como que… te da esa seguridad, de decir fallé una vez y ahora qué hago, a lo mejor me dejan sola, no, no te dejan sola, porque me dice “ya empezamos a bailar, vamos a seguir bailando hasta que se termine la canción”, y es así.” (Age 33, Accompaniment) | “What I always felt was that sense of security, you are not going to come out of this alone, we are never going to abandon you, this is where we solve this. That kind of...gives you that sense of security, to say I failed once and now what do I do, maybe they will leave me on my own, no, they will not leave you alone, because they told me, ‘we already started to dance, we are going to continue dancing until the song is over’, and that is how it is.” (Age 33, Accompaniment) |
| “Me sentí…cómo te puedo decir, muy bien atendida, no esperaba yo llegar acá, muy contenida emocionalmente…” (Age 26, Clinic). | “I felt...how can I say it, very well attended to, I did not expect to get here, very emotionally *contenida*...” (Age 26, Clinic). |
| “Sentí… me sentí liviana, me sentí apoyada, acompañada, y sabía que todo iba a estar bien, entonces más tranquila me sentí, mucho más tranquila, porque estaba muy asustada, sentí que estaba mi problema resuelto, eso sentí” (Age 26, Accompaniment). | “I felt...I felt light, I felt supported, *acompañada*, and I knew that everything would be fine, so I felt calmer, much calmer, because I was very scared, I felt that my problem was solved, that is what I felt.” (Age 26, Accompaniment). |

**Theme:** **Attentive communication and interactions**

| **Spanish** | **English** |
| --- | --- |
| “Bien, no me puedo acordar el nombre de la chica que estaba acompañándome, pero bien, sentada al lado mío, hablándome, como si fuera que nos conocíamos de siempre, la médica también, que estaba, me iba contando todo lo que iba haciendo. Que iba re bien todo. Siempre preguntándome si me dolía, si qué sentía, si sentía algo raro.”(Age 25, Clinic) | “Well, I can’t remember the name of the girl who was accompanying me, but well, she was sitting next to me, talking to me, as if we knew each other forever, the doctor too, who was there, was telling me everything she was doing. That everything was going well. Always asking me if it hurt, what I felt, if I felt something strange.”(Age 25, Clinic) |
| “Muy atentas ellas porque me decían: ‘te voy a poner esto, te voy a poner aquello, te voy a tocar acá.’ Entonces cosas que vos no estás acostumbrada tampoco a que suceda. Ninguna especialista te va a decir permiso.” (Age 41, Clinic). | “They were very attentive because they told me, ‘I’m going to put this on you, I’m going to put that on you, I’ll touch you here.’ So these are things that you are not accustomed to happening either. No specialist is going to ask you for your permission” (Age 41, Clinic). |
| “Desde el día que vos lo vas a hacer ya te están acompañando, o sea, no es que te muestran cómo hacer…hay un antes, el durante y el después, y todo es con acompañamiento, y eso está re bueno, porque vos sabés que en el momento si te pasa algo no es que vos te vas a un hospital o algo, vos tenés a quien consultar, quién te puede ayudar.” (Age 30, Accompaniment) | “From the day you are going to do it, they are already accompanying you, that is, it is not that they are just going to show you how to do it… there is a before, during and after, and everything is with *acompañamiento*, and that is really good, because you know that in the moment if something happens to you it is not that you go to a hospital or something, you have someone to consult, who can help you.” (Age 30, Accompaniment) |
| “Pero fue como… como muy aliviante y sentí la contención más allá de los kilómetros y más allá del no contacto cara a cara, sentí mucho la contención y sabía que estaba, no fue la única vez que me llamó, me llamó ahí, me explicó todo, me llamó antes, después del proceso, y durante también. (Age 30, Accompaniment) | “But it was like… it was very relieving and I felt the *contención* beyond the kilometers and beyond the non-contact face to face, I felt the *contención* and I knew they were there, it was not the only time they called me…, they explained everything to me, they called me before, after the process, and during the process too.” (Age 30, Accompaniment) |

**Theme:** **Comprehensive, clear information provision**

| **Spanish** | **English** |
| --- | --- |
| “Explicó todo paso por paso, y era todo como ‘sí, dale, quiero interrumpir acá’, y nada, en cuanto a contención acá encontré el 100%, o sea, contención que no encontraba en él, que no encontraba en mi amiga, en el psicólogo” (Age 20, Clinic). | “They explained everything step by step, and it was all like, ‘yes, let’s go, I want to end [my pregnancy] here’, and nothing, in terms of *contención* here I found it 100%, that is, *contención* that I did not find in him [my partner], that I did not find in my friend, in the psychologist.” (Age 20, Clinic) |
| “Esto de que sea grupal te hace sentir un poco más relajado, porque vos sabés que no sos la única y que por ahí otras van a hacer preguntas que a vos no se te ocurren y que te puede llegar a pasar, entonces como que estás más segura de lo que estás haciendo.” (Age 30, Accompaniment). | “This thing of it being in a group makes you feel a little more relaxed, because you know that you are not alone and that there others are going to ask questions that do not occur to you and that could happen to you, so, then you are like more sure of what you are doing.” (Age 30, Accompaniment). |
| “Como que te hablaba de una manera que vos podías llegar a entender todo, ¿entendés? Y yo que vine sin saber nada… la verdad es que me inspiró tanta confianza, tanta seguridad, me explicó todo, absolutamente todo.” (Age 32, Clinic). | “It was like they spoke to you in a way that you could understand everything, you understand? And I came without knowing anything...the truth is that they inspired so much confidence in me, so much security, they explained everything, absolutely everything.” (Age 32, Clinic). |

**Theme: Empathetic and non-judgmental listening**

| **Spanish** | **English** |
| --- | --- |
| “Te contienen emocionalmente, te escuchan, te saben escuchar, te saben entender la situación que estás pasando y profesionalmente son muy delicados, respetuosos, atentos...” (Age 26, Clinic). | “They provide *contención* emotionally, they listen to you, they know how to listen, they know how to understand the situation you are going through and professionally they are very delicate, respectful, attentive.” (Age 26, Clinic) |
| “Ya sé que pasan un montón de chicas por acá, pero como que te hacían sentir, no sé, no especial pero como que importaba lo que vos estés sintiendo, y que estaba bien tipo tu decisión, sea cual sea, que importaban los motivos por los que vos no querías tenerlo.”(Age 20, Clinic) | “I know that a lot of girls pass through here, but they like made you feel, I don't know, not special, but like it mattered what you were feeling, and that your decision was fine, whatever it was, that the reason why you didn’t want to have it [the pregnancy] mattered.” (Age 20, Clinic) |
| “Como que acá [aborto] está naturalizado, no es un enjuiciamiento que se hace” (Age 33, Accompaniment). | “Since here [abortion] is normalized, what we do is not judged in any way” (Age 33, Accompaniment). |
| “por qué tengo que ir a una entrevista colectiva? Primero no me gustó un carajo y después yo entendí por qué era colectiva, o por lo menos yo me lo expliqué a través de la experiencia que tuvimos, y eso me gustó… medio como que está bien pensado desde el lugar este de sacarlo de lo oscuro o clandestino o práctica ilegal o a escondidas, el hecho de hacerlo colectivo.” (Age 36, Clinic) | “‘Why do I have to go to a collective interview?’ At first I didn’t like that one bit and after, I understood why it was collective, or at least I explained it to myself through the experience we had, and I liked that...it’s like half because it’s good thinking from the perspective of taking it [abortion] out of the dark or the clandestine or illegal practice or secret practice, the fact that it is collective.” (Age 36, Clinic) |
| “Las chicas lo hacían tipo más ameno, parecía una charla de amigas. Sí, estuvo re bueno. Sí, me sentí muy bien, contenida, estuvo bueno” (Age 30, Accompaniment). | “The girls made it more enjoyable, it seemed like a chat with friends. Yes, it was really good. Yes, I felt very good, I felt *contenida*, it was good” (Age 30, Accompaniment) |

**Theme: Choice in pain management**

| **Spanish** | **English** |
| --- | --- |
| “Ellos me pusieron música, Los Redondos, y la verdad es que te soy sincera, yo no sentí ningún dolor…las chicas me hablaban y charlábamos, y estaba con la bolsa de agua caliente como por si sentís dolor.” (Age 32, Clinic) | “They put music on for me, Los Redondos [a band], and the truth is that if I’m honest with you, I didn’t feel any pain…the girls talked to me and we chatted, and I had the hot water bottle, for like in case you feel pain.” (Age 32, Clinic) |
| “Me dijo algo que me sorprendió mucho, me dijo ‘si querés poner música’, que me sorprendió para bien en el sentido de que…a qué nivel están, no sé cómo decirlo, como pensando en la comodidad de que el paciente realmente un momento así lo pase lo mejor posible.” (Age 21, Clinic). | “They told me something that really surprised me, they said ‘if you want, we can play music’, which surprised me in the best sense...at what level they are, I don’t know how to say it, as if they are really thinking about the comfort of the patient, in a moment like that, I had the best time possible.” (Age 21, Clinic) |
| “Ella [la acompañante] me dijo que esté tranquila, que cuanto más tranquila esté iba a ser más rápido y más fácil, me dijo genérate un lindo ambiente, que la persona que esté con vos te transmita cosas buenas, si te gustan los sahumerios prendé un sahumerio, prepárate lo que te guste para comer…, me hice una tarta de dulce de leche, nueces y chocolate… me dijo mirá lo que quieras, lo hice mirando Ru Paul.”(Age 30, Accompaniment) | “She [the accompanier] told me to stay calm, that the calmer I was, the faster and easier it would be, she told me to create a nice ambiance, that the person who is with you transmits good things to you, if you like incense, light an incense stick, prepare whatever you like to eat…I made myself a cake of dulce de leche [caramel], walnuts and chocolate…she told me watch whatever you want, so I did it watching Ru Paul [television show]” (Age 30, Accompaniment) |
