# Supplementary material for: Perspectives on high-quality interpersonal care among people obtaining abortions in Argentina
Source: Reprod Health. 2022 May 2;19:107. doi: 10.1186/s12978-022-01401-1 (PMC9059438; doi:10.1186/s12978-022-01401-1)
Supplement: Supplementary file 3 — Additional file 3. Spanish version of article. [file 12978_2022_1401_MOESM3_ESM.docx]

**Perspectivas sobre la atención personalizada de alta calidad entre personas que obtienen un aborto en Argentina**

Chiara Bercu^1^, Sofia Filippa^1^ Ana Maria Ramirez^1^, Anna Katz^1^, Belén Grosso^2^, Ruth Zurbriggen^2^, Sandra Vázquez^3^, Sarah E. Baum^1^

*^1^Ibis Reproductive Health, Oakland, CA;  ^2^ Colectiva Feminista La Revuelta, Neuquén - Patagonia, Argentina;* ^3^*Grupo FUSA Asociación Civil Buenos Aires, Argentina*

**Autor para correspondencia:** Chiara Bercu, cbercu@ibisreproductivehealth.org

**Palabras clave:** aborto, acompañamiento, salud reproductiva, atención centrada en la persona, calidad de la atención, Argentina

**Declaraciones**

**Aprobación ética y consentimiento para participar:** Este estudio fue aprobado por el *Allendale Investigational Review Board* (Comité de Revisión de Investigación Allendale) con sede en los Estados Unidos y por el Comité de Bioética de la Fundación Huésped, con sede en Argentina. Todas las participantes proporcionaron un consentimiento verbal para participar en el estudio.

**Consentimiento para la publicación:** No corresponde.

**Disponibilidad de los datos y materiales:** Los conjuntos de datos que se generaron y analizaron durante este estudio no están disponibles al público, para mantener la confidencialidad y reducir los riesgos para las participantes. La guía para la entrevista se publica como un apéndice.

**Intereses en conflicto:** Las autoras declaran que no tienen intereses en conflicto.

**Financiamiento:** El financiamiento para este trabajo fue proporcionado por la Fundación David and Lucile Packard Foundation y la Fundación Children’s Investment Fund Foundation. Los patrocinadores no tuvieron ningún rol en el diseño, redacción ni decisión de publicación.

**Contribuciones de las autoras:** CB y SF contribuyeron al diseño, codificación, análisis y redacción del manuscrito del estudio. AR y AK contribuyeron al desarrollo del libro de códigos y al diseño del estudio, y revisaron los borradores preliminares del manuscrito. BG & RZ contribuyeron al diseño del estudio, al reclutamiento, la recopilación de los datos, a la interpretación de los hallazgos, y revisaron los borradores preliminares del manuscrito. SV contribuyó con el reclutamiento, la interpretación de los hallazgos y revisó los borradores preliminares del manuscrito. SB contribuyó con el diseño, la codificación, el análisis y la redacción del manuscrito del estudio. Todas las autoras leyeron y aprobaron el manuscrito final.

**Agradecimientos:** Quisiéramos agradecer a nuestra colega, María Victoria Simón, por colaborar con la recopilación de los datos en Casa FUSA. También quisiéramos agradecer a todos los miembros de Colectiva Feminista La Revuelta y de Casa FUSA que hicieron posible este estudio. Por último, agradecemos las/os investigadores asociadas/os de Abortion Service Quality, Ipas y Metrics for Management por sus contribuciones al diseño y conceptualización del estudio.

**Resumen conciso**

Poco se sabe acerca de cómo las personas que abortan perciben la calidad de la atención que reciben y de qué aspectos de las interacciones personales con los/las proveedores/as son importantes para ellas. La finalidad de este estudio cualitativo fue comprender las preferencias de las clientes de aborto de una clínica de salud reproductiva y de un grupo de acompañamiento en Argentina. Entrevistamos a 24 personas que obtuvieron atención para abortar en Buenos Aires y en Neuquén, Argentina. Las consultamos acerca de sus experiencias y sus preferencias de atención del aborto. Luego, analizamos las entrevistas y evaluamos de manera específica los aspectos de atención interpersonal con base en marcos de calidad de atención que se encuentran en la literatura.

Las participantes describieron a la atención del aborto de alta calidad como una sensación de recibir acompañamiento y contención de sus proveedores/as, términos que implican recibir una atención amable, cariñosa, compasiva y de contención emocional durante sus abortos. Describieron cuatro elementos clave de las interacciones interpersonales: la comunicación atenta de los/las proveedores/as y acompañantes, la provisión de información clara y comprensible, el apoyo sin juzgamiento y las opciones individualizadas para el manejo del dolor.

En conclusión, las personas que obtuvieron abortos en dos modelos de atención diferentes en Argentina identificaron de manera consistente al hecho de recibir una atención compasiva y contenedora durante el aborto como uno de los aspectos clave de la atención personalizada. Los hallazgos tienen implicancias para la incorporación de las perspectivas de las personas que abortan en el desarrollo de directrices de atención, la capacitación de proveedores/as, y la supervisión y mejora de los servicios. Esto tiene particular importancia ya que el gobierno de Argentina se prepara para expandir el acceso legal al aborto.

**Resumen**

**Introducción:** Poco se sabe acerca de cómo las personas que abortan describen la atención personalizada de alta calidad en Argentina. La finalidad de este estudio cualitativo fue comprender las preferencias y las prioridades en sus interacciones interpersonales con proveedores/as.

**Diseño del estudio:** Realizamos 24 entrevistas en profundidad a personas que obtuvieron atención para abortar en Buenos Aires y en Neuquén, Argentina. Usamos el enfoque de análisis temático con base en los dominios interpersonales presentes en los marcos actuales de calidad de la atención para codificar las transcripciones de manera iterativa.

**Resultados:** Quienes participaron describieron a la atención del aborto de alta calidad como una sensación de recibir acompañamiento y contención de sus proveedores/as, términos que implican recibir una atención amable, cariñosa, compasiva y de contención emocional durante sus abortos. Describieron cuatro elementos clave de las interacciones interpersonales: la comunicación atenta de los/las proveedores/as y acompañantes, la provisión de información clara y comprensible, el apoyo sin juzgamiento y las opciones individualizadas para el manejo del dolor.

**Conclusiones:** Las personas que obtuvieron abortos en dos modelos de atención diferentes en Argentina identificaron de manera consistente al hecho de contar con una atención compasiva y contenedora durante el aborto como uno de los aspectos clave de la atención. Los hallazgos tienen implicancias para la incorporación de las perspectivas de las personas en el desarrollo de directrices de atención, la capacitación de proveedores/as, y la supervisión y mejora de los servicios. Esto tiene particular importancia ya que el gobierno de Argentina se prepara para expandir el acceso legal al aborto.

1. **Antecedentes**

En Argentina, el aborto estuvo legalmente restringido y regulado por el Código Penal hasta enero de 2021. En enero de 2021, se promulgo una ley que despenaliza y legaliza el aborto voluntario hasta las 14 semanas de gestación sin restricciones de causales^1^. Antes de este cambio en la legislación, cuando se realizó esta investigación, el aborto legal solo estaba disponible si el embarazo ponía en riesgo la vida o la salud de la persona embarazada^[[1]](#footnote-1)^ o si el embarazo era el resultado de una violación. Si bien algunas provincias del país garantizaban la interrupción legal del embarazo en los casos en que llevar el embarazo hasta su término ponía en riesgo el bienestar físico, emocional o social de la persona, muchas provincias no lo garantizaban. A pesar de existir el aborto legal por causales, numerosas barreras impidieron que las personas accedieran a la atención, incluso la falta de conocimiento con respecto a la ley, proveedores/as que se negaron a proporcionar los servicios, y el estigma y las creencias normativas con respecto al aborto^2,3^. Los servicios de aborto se proporcionaban a través de un rango de modelos de atención en Argentina, incluso de clínicas y hospitales de salud reproductiva integral que proporcionaban servicios legales de aborto y de grupos de acompañamiento que ofrecían información y apoyo presencial y virtual para personas de todo el país que estaban autogestionando un aborto con medicamentos^4,5^. A pesar de las barreras legales y sociales que existían para el acceso al aborto antes de enero de 2021, el aborto en Argentina era común y las personas tenían abortos seguros y eficaces en todos los modelos de atención. Sin embargo, poco se sabe acerca de cómo las personas percibieron la calidad de los servicios de aborto que recibieron y de lo que era importante para ellas en sus interacciones con los/las proveedores/as.

La investigación existente sobre la calidad de la atención del aborto ha enfatizado la importancia de la atención interpersonal; la atención interpersonal se refiere a las experiencias y percepciones que tienen las personas con respecto a la interacción con la atención médica^6^. Una revisión sistemática de los indicadores publicados para medir la calidad de los servicios de aborto concluyó que las mediciones se enfocaban, en gran parte, en los aspectos estructurales y del proceso, como infraestructura y capacidad técnica, y que solo un tercio de los indicadores había incorporado las preferencias de las/los clientes en su desarrollo.^7^ No se han incluido de manera consistente indicadores que midan la atención interpersonal; en consecuencia, existen brechas en nuestra comprensión de qué aspectos de la interacción cliente-proveedor son más importantes para las personas que reciben servicios^8,9^. La *Interpersonal Quality in Abortion Care* (Escala de calidad interpersonal en la atención del aborto) se desarrolló en 2019 con la intención de cubrir esta brecha al crear una medición que deriva de las perspectivas de las personas en los Estados Unidos; la escala incluye elementos que se relacionan con el respeto, la sensación de ser escuchada/o y la amabilidad^8^. Estos dominios enfatizan la importancia de las interacciones interpersonales en la atención centrada en la persona.

Se define a la atención centrada en la persona como la atención que está orientada en las necesidades, expectativas y preferencias identificadas de la persona recibiendo servicios^10^. La atención centrada en la persona se basa en las interacciones interpersonales entre las personas y sus proveedores/as, y la capacidad de los/las proveedores/as de centrarse en las necesidades de las personas que atienden durante la atención. Los estudios conducidos entre mujeres que también abordan la atención centrada en la persona en servicios de salud anticonceptiva y materna demostraron que ellas valoran factores interpersonales como la confianza, la empatía, el respeto, el apoyo, la confidencialidad y la atención sin juzgamiento,^11–14^ y que estos dan forma a las percepciones de calidad y al comportamiento de búsqueda de atención para servicios médicos en el futuro^15–17^. Existe evidencia más reciente que surge dentro del contexto del aborto y demuestra cómo las experiencias de calidad de las personas se ven afectadas de manera positiva por un enfoque centrado en la persona^18^, como recibir consejería integral, atención sin juzgamiento y atención contenedora, al igual que una sensación de autonomía y libertad de elección^18–20^. Si bien la cantidad de estudios en América Latina que se enfocan en las perspectivas de las personas sobre la calidad de aborto es limitada, estos han encontrado que las interacciones cliente-proveedor y la provisión de información se identifican como algunos de los aspectos más sobresalientes de las experiencias de las/los pacientes^21,22^. Un estudio sobre la calidad de la atención postaborto en hospitales públicos en Argentina concluyó que, por lo general, no se proporcionaba atención de manera respetuosa y que no estaban presentes aspectos clave, como la provisión de información.^22^

A pesar de que sabemos la importancia que tiene la atención interpersonal en la calidad de la atención, existen brechas en la comprensión de las preferencias y las prioridades entre las personas que buscan un aborto en diferentes contextos y modelos de atención, en particular, fuera de los sistemas de salud. Analizamos entrevistas con personas que recibieron atención para abortar ya sea en una clínica de salud reproductiva integral o a través de un grupo de acompañamiento, con el objetivo de lograr una comprensión más profunda sobre cómo las personas que obtienen un aborto experimentan las interacciones con los/las proveedores/as y qué aspectos de la atención interpersonal les parecen valiosos. Este análisis es particularmente oportuno dado el cambio en la legislación que permite el aborto hasta las 14 semanas; esperamos que estos datos cualitativos puedan informar a la integración de la atención centrada en la persona durante la implementación de la atención para interrupción voluntario del embarazo en todo el país.

1. **Métodos**

Las entrevistas que se analizaron en este manuscrito fueron parte de un estudio más grande que se realizó en cuatro países (Argentina, Bangladesh, Etiopia y Nigeria), que tuvo la finalidad de lograr una comprensión más profunda de las experiencias de las personas ^[[2]](#footnote-2)^ con los servicios de aborto y sus percepciones de la calidad de la atención^23^. Para este manuscrito, analizamos entrevistas en profundidad semiestructuradas con personas que obtuvieron un aborto en Buenos Aires y en Neuquén, Argentina.

Las participantes fueron reclutadas de dos organizaciones diferentes que ofrecen atención para abortar: a través de un grupo feminista de acompañamiento con sede en Neuquén y de una clínica de salud reproductiva integral en Buenos Aires. El grupo feminista de acompañamiento, Colectiva Feminista, La Revuelta (La Revuelta) realiza sesiones informativas grupales en las que las personas que buscan un aborto aprenden cómo tener un aborto autogestionado seguro y cómo tener un aborto autogestionado seguro con medicamentos (AM) en el hogar. La Revuelta está compuesta por voluntarias/os capacitadas/os, o “acompañantes”, que proporcionan apoyo e información con base en evidencias, ya sea presencial o por teléfono, durante el proceso de aborto con medicamentos a las personas que buscan un aborto. En este documento, se usa el término “acompañantes” para referirse a las activistas que forman parte de La Revuelta, que proporcionan apoyo e información sobre el aborto. La clínica de salud reproductiva Casa FUSA, Centro para Atención de Adolescentes y Jóvenes (FUSA), es un establecimiento de atención privada que proporciona atención del aborto en una clínica en Buenos Aires. Si bien FUSA ofrece también aborto con medicamentos, para esta investigación solo reclutamos personas que habían recibido una aspiración manual (AMEU), una práctica común para el aborto inducido o para la atención postaborto^24^. En nuestro estudio, las personas que se realizaron su aborto con FUSA visitaron la clínica antes del aborto para participar en una sesión informativa grupal y en una sesión de consejería individual con un/a proveedor/a, y luego regresaron a los pocos días para su AMEU. Durante la sesión informativa grupal, se les proporcionó a las/los clientes información acerca del aborto en general, la situación legislativa del aborto, los métodos de aborto y su seguridad y eficacia, mientras que la sesión de consejería individual fue un espacio para que las participantes hablaran con los/las proveedores/as acerca de cualquier inquietud individual que no se hubieran sentido cómodas para plantear en la sesión grupal. En este documento, el término “proveedores/as” se refiere tanto al personal médico como al personal en general que proporciona atención del aborto en FUSA. Dado que los centros de reclutamiento de este estudio eran operados por activistas feministas y organizaciones sin fines de lucro, es probable que los servicios que proporcionaron hayan sido de mejor calidad que los del promedio de los centros privados y públicos en Argentina.

Realizamos 24 entrevistas en profundidad semiestructuradas, 12 en cada centro de reclutamiento, entre noviembre de 2018 y enero de 2019. El tamaño de la muestra se determinó como parte del estudio más grande. Nuestra intención era realizar aproximadamente 100 entrevistas, divididas entre cuatro países en partes iguales. En Argentina, decidimos dividir las entrevistas en partes iguales entre ambos modelos de atención. Personal capacitado de cada centro convocó a las participantes durante una sesión informativa grupal antes del aborto o por teléfono luego de que el aborto se hubiera completado. Todas las entrevistas se realizaron luego de que se completara el aborto. Para ser elegibles para el estudio, las participantes tenían que ser mayores de 15 años, poder proporcionar un consentimiento informado, poder hablar español, y haber tenido un aborto con La Revuelta o FUSA dentro de los 6 meses previos a la convocatoria para el estudio.

Una entrevistadora (BG) realizó todas las entrevistas en persona en Neuquén y Buenos Aires. BG se identifica como mujer y tiene vasta capacitación académica y experiencia en la realización de investigación ética, en métodos cualitativos y en técnicas de entrevistas cualitativas. Todas las entrevistas se realizaron en un espacio proporcionado por La Revuelta o FUSA, o en un lugar seguro elegido por quien participo. Para mantener la privacidad de participantes, dada la sensibilidad del tema, las únicas personas presentes fueron BG y la persona entrevistada. Luego de cada entrevista, BG realizó notas de campo. BG estaba asociada con La Revuelta al momento del estudio, pero no oficiaba de acompañante ni conocía previamente a ninguna de las personas que se reclutaron para participar.

La guía para la entrevista se desarrolló para el estudio más grande con base en marcos de calidad de la atención^9,25,26^ al igual que en estudios previos sobre calidad del aborto. Se realizó un piloto del estudio y se lo adaptó al contexto argentino, para garantizar la claridad o aplicabilidad cultural. La guía para la entrevista incluyó preguntas sobre las experiencias de aborto de la persona que abortó y también de las interacciones con proveedores/as (Véase el Archivo complementario I: Guía para la entrevista en español). Las entrevistas duraron entre 40 y 60 minutos y se grabaron en audio. Las personas entrevistadas participantes fueron recompensadas con una tarjeta de transporte público por un valor aproximado de $10 USD. Este estudio fue aprobado por el *Allendale Investigational Review Board* (Comité de Revisión de Investigación Allendale) con sede en los Estados Unidos y por el Comité de Bioética de la Fundación Huésped, con sede en Argentina.

Realizamos un análisis temático para explorar las percepciones y las experiencias que tenían las personas con respecto a la atención interpersonal durante sus abortos. Todas las entrevistas fueron transcriptas por un profesional y se las analizó temáticamente en español. Desarrollamos un libro de códigos inicial mediante el uso de temas clave a partir de la guía para la entrevista, los dominios de calidad de la atención extraídos de una revisión de la literatura sobre el tema, y los códigos que emergieron de las transcripciones. Dos miembros del equipo de investigación (CB y SF) realizaron una doble codificación de dos transcripciones, luego de lo cual se reunieron para discutir las discrepancias en la codificación y ajustaron, redefinieron o modificaron los códigos y las descripciones de los códigos, y luego aplicaron el libro de códigos a las 24 transcripciones. Usamos MAXQDA 2018 (Software VERBI, 2019) para la codificación. Analizamos los temas que emergieron durante el proceso de redacción de los resúmenes de los códigos y evaluamos patrones y trazamos las tendencias dentro del conjunto de datos. El tamaño de nuestra muestra no fue suficiente para realizar un análisis comparativo entre los dos modelos de atención, por lo tanto, analizamos el rango de experiencias en todo el conjunto de datos. Cuando se observaron diferencias en la provisión de la atención con base en el modelo de atención, incluimos estas diferencias en los resultados. Personal clave de los centros de reclutamiento colaboró con la interpretación de los datos mediante la revisión de los hallazgos preliminares. Las citas presentadas en este manuscrito se presentan en su idioma original, español.

1. **Resultados**

Entre las 24 personas que participaron, la edad promedio fue de 30 años, con un rango de entre 20 y 41 años de edad. Un cuarto informó haber tenido un aborto después de 12 semanas de gestación. La mitad tuvo un aborto con medicamentos (todas reclutadas del modelo de acompañamiento), y la otra mitad tuvieron una aspiración manual (AMEU) (todas reclutadas de la clínica). Hubo seis participantes que informaron haber tenido un aborto previo y nueve que ya tenían hijos. Ninguna de las participantes informó estar casada, sin embargo, casi la mitad informó estar en una relación, cuatro de las cuales vivían con su pareja. Diez participantes informaron tener un empleo pago, cinco informaron ser estudiantes a tiempo completo, seis estudiaban y tenían un empleo pago y tres informaron estar desempleadas al momento de la entrevista. *(Tabla 1)*

***Tabla 1: Características de las participantes***

|  | **N=24**  **n (%)** |
| --- | --- |
| **Edad (años)** | |
| Mediana | 30 |
| Rango | 20-41 |
| **Método de aborto** | |
| Aborto con medicamentos | 12 (50,0 %) |
| Aspiración manual (AVM) | 12 (50,0 %) |
| **Edad gestacional** | |
| <=12 semanas | 16 (66,6 %) |
| >12 semanas | 6 (25,0 %) |
| Sin datos | 2 (8,3 %) |
| **Aborto previo** | |
| Sí | 6 (25,0 %) |
| No | 18 (75,0 %) |
| **Hijos** | |
| Sí | 9 (37,5 %) |
| No | 15 (62,5 %) |
| **Estado civil** | |
| Casada/o | 0 (0,0 %) |
| En una relación | 11 (45,8 %) |
| Soltera/o | 13 (54,2 %) |
| **Situación laboral** |  |
| Empleada/o | 10 (41,7 %) |
| Estudiante | 5 (20,8 %) |
| Empleada/o y estudiante | 6 (25,0 %) |
| Desempleado/a | 3 (12,5 %) |

***Acompañamiento* y *Contención***

En su totalidad, quienes participaron tanto de la clínica como de los modelos de atención con acompañamiento describieron a la atención del aborto de alta calidad como una sensación de recibir de acompañamiento y contención de sus proveedores/as. Estos términos emergieron una y otra vez en las descripciones de las interacciones positivas, y en toda la muestra se hizo hincapié en que era necesaria para una buena atención. La atención interpersonal proporcionada con acompañamiento y contención ayudó a que las participantes confiaban en su proveedor/a o acompañante, quienes, a su vez, reaseguraron su sensación de seguridad durante el aborto. Al describir su experiencia con La Revuelta, una participante explicó, *“[Y]o siempre digo que te brindan una seguridad que por ahí capaz que en otro lugar no sé si… a ver, no son médicos, pero el hecho de la contención es re importante, no te sentís sola” (Edad 34, Acompañamiento)*. Otra participante, que no experimentó ningún síntoma de aborto después de tres intentos de aborto con medicamentos, enfatizó la importancia de saber que no sería abandonada. Con el apoyo de su acompañante, la participante, en última instancia, terminó recibiendo una AMEU en una clínica;

*“Lo que sí siempre sentí como esa seguridad, de esta no vas a salir sola, no te vamos a abandonar jamás, de esta solucionamos esto. Eso como que… te da esa seguridad, de decir fallé una vez y ahora qué hago, a lo mejor me dejan sola, no, no te dejan sola, porque me dice “ya empezamos a bailar, vamos a seguir bailando hasta que se termine la canción”, y es así.” (Edad 33, Acompañamiento)*

El acompañamiento y la contención también desempeñaron un papel en el alivio de las ansiedades de las participantes. Las participantes describieron una sensación de alivio al darse cuenta de podrían interrumpir sus embarazos en un contexto de atención de alta calidad. Una participante describió; *“Me sentí…cómo te puedo decir, muy bien atendida, no esperaba yo llegar acá, muy contenida emocionalmente…”* *(Edad 26, Clínica)*. Por último, una participante expresó sentirse “liviana” después de reunirse con el grupo de acompañamiento, ya que sabía que las acompañantes la ayudarían a interrumpir su embarazo; *“Sentí…me sentí liviana, me sentí apoyada, acompañada, y sabía que todo iba a estar bien, entonces más tranquila me sentí, mucho más tranquila, porque estaba muy asustada, sentí que estaba mi problema resuelto, eso sentí” (Edad 26, Acompañamiento).*

A continuación, presentamos cuatro aspectos clave de la atención del aborto con acompañamiento y contención, que ayudaron a definir lo que las participantes en Argentina valoraron durante sus interacciones interpersonales: la comunicación atenta de los/las proveedores/as y acompañantes, la provisión de información clara y comprensible, el apoyo sin juzgamiento y las opciones individualizadas para el manejo del dolor.

**Comunicación e interacciones atentas**

Muchas participantes en el estudio describieron sentirse apoyadas durante las interacciones interpersonales, ya que los/las proveedores/as y acompañantes se comunicaron continuamente y estuvieron atentos/as a sus necesidades durante todo su proceso de aborto, en particular, durante el procedimiento de aborto en sí. Una participante en la clínica valoró el hecho de que la proveedora le explicara cada paso de su AMEU a medida que ocurría, y se mostró muy atenta a su comodidad;

*“Bien, no me puedo acordar el nombre de la chica que estaba acompañándome, pero bien, sentada al lado mío, hablándome, como si fuera que nos conocíamos de siempre, la médica también, que estaba, me iba contando todo lo que iba haciendo. Que iba re bien todo. Siempre preguntándome si me dolía, si qué sentía, si sentía algo raro.” (Edad 25, Clínica)*

Cuando los/las proveedores/as y acompañantes demostraron preocupación por su bienestar físico y emocional, las participantes se sintieron más seguras, más calmadas y confiaron más en el/la proveedor/a o acompañante. Una participante resaltó el hecho de que su proveedora le pidió permiso para tocarla antes de comenzar el procedimiento de AMEU, algo que ella no esperaba. Esto ayudó a que sintiera que estaba recibiendo una atención respetuosa; ella explica;

*“Muy atentas ellas porque me decían: ‘te voy a poner esto, te voy a poner aquello, te voy a tocar acá.’ Entonces cosas que vos no estás acostumbrada tampoco a que suceda. Ninguna especialista te va a decir permiso.” (Edad 41, Clínica).*

Las participantes que fueron acompañadas por el grupo de acompañamiento feminista, describieron haber sentido contención y acompañamiento durante sus interacciones con su acompañante, desde el principio al fin de la experiencia. Esta participante explicó la importancia de saber que la acompañante estaría allí para ella si algo pasaba;

*“Desde el día que vos lo vas a hacer ya te están acompañando, o sea, no es que te muestran cómo hacer…hay un antes, el durante y el después, y todo es con acompañamiento, y eso está re bueno, porque vos sabés que en el momento si te pasa algo no es que vos te vas a un hospital o algo, vos tenés a quien consultar, quién te puede ayudar.” (Edad 30, Acompañamiento)*

En algunos casos, las participantes se sorprendieron por cuán apoyadas se sintieron en las interacciones que tuvieron con las acompañantes por teléfono y por mensajes de texto, como, por ejemplo, esta participante;

*“Pero fue como… como muy aliviante y sentí la contención más allá de los kilómetros y más allá del no contacto cara a cara, sentí mucho la contención y sabía que estaba, no fue la única vez que me llamó, me llamó ahí, me explicó todo, me llamó antes, después del proceso, y durante también”.* *(Edad 30, Acompañamiento)*

La sensibilidad que acompañantes y proveedores/as les demostraron a quienes abortaron ayudó a que sintieran confianza en esas personas, también sintieron cuidados y apoyo durante sus abortos.

**Provisión de información clara y completa**

Casi todas las personas entrevistadas describieron la importancia de recibir información clara e integral de sus proveedores/as o acompañantes. En ambos espacios, la información integral incluyó detalles de cada paso del proceso de aborto y se proporcionó durante una sesión informativa grupal, como explica esta persona;

*“Explicó todo paso por paso, y era todo como ‘sí, dale, quiero interrumpir acá’, y nada, en cuanto a contención acá encontré el 100%, o sea, contención que no encontraba en él, que no encontraba en mi amiga, en el psicólogo”.* *(Edad 20, Clínica)*

Para esta participante, la información detallada que recibió sobre el procedimiento la ayudó a decidir tener su aborto en la clínica. Además, esta participante vinculó a la provisión de información con una sensación de contención en la clínica y con tener una sensación de apoyo que no había sentido ni recibido de otras personas. Esto enfatiza cómo el hecho de proporcionar transparencia acerca del procedimiento de aborto podría haber facilitado una sensación de confianza entre las personas y los/las proveedores/as.

Quienes participaron también valoraron la oportunidad de realizar preguntas y hablar acerca de sus circunstancias con los/las proveedores/as y acompañantes, y también con otras personas que estaban allí para realizarse un aborto. Una participante mencionó que se les dio la oportunidad de interactuar con los instrumentos de AMEU antes del procedimiento en el modelo clínico. Además, la sesión informativa grupal que usaron ambos modelos ofreció un espacio para que escuchen las preguntas que tenían otras personas y les infundió una sesión de comunidad y seguridad. Como explicó una participante;

*“Esto de que sea grupal te hace sentir un poco más relajado, porque vos sabés que no sos la única y que por ahí otras van a hacer preguntas que a vos no se te ocurren y que te puede llegar a pasar, entonces como que estás más segura de lo que estás haciendo.” (Edad 30, Acompañamiento)*

Varias personas enfatizaron la importancia de recibir información que estaba fácilmente accesible y era comprensible, como explica esta participante:

*“Como que te hablaba de una manera que vos podías llegar a entender todo, ¿entendés? Y yo que vine sin saber nada… la verdad es que me inspiró tanta confianza, tanta seguridad, me explicó todo, absolutamente todo.” (Edad 32, Clínica)*

El hecho de recibir información exhaustiva ayudó a que las personas que buscaron un aborto se sintieran seguras, a que confiaran en sus proveedores/as y a sentirse bien preparadas para sus experiencias de aborto.

**Escucha empática y no crítica**

Las personas entrevistadas a menudo mencionaron la importancia de sentirse escuchadas por sus proveedores/as o acompañantes. Esto hizo que se sintieran respetadas y comprendidas. Una participante explicó, *“Te contienen emocionalmente, te escuchan, te saben escuchar, te saben entender la situación que estás pasando y profesionalmente son muy delicados, respetuosos, atentos...” (Edad 26, Clínica).* Otra participante describió cómo se sintió al poder compartir sus motivos para querer un aborto con un/a proveedor/a que la ayudó a sentir que su experiencia era importante;

*“Ya sé que pasan un montón de chicas por acá, pero como que te hacían sentir, no sé, no especial pero como que importaba lo que vos estés sintiendo, y que estaba bien tipo tu decisión, sea cual sea, que importaban los motivos por los que vos no querías tenerlo.” (Edad 20, Clínica)*

El hecho de percibir que los/las proveedores/as y acompañantes no estigmatizaban ni emitían ningún juicio con respecto a su decisión, sino que escuchaban de manera empática, ayudó a las personas entrevistas a sentir que estaban recibiendo atención con acompañamiento y contención.

También relataron que, en ambos modelos de atención, sintieron que los/las proveedores/as se esforzaron por naturalizar el aborto. Una participante explica acerca del modelo de acompañamiento; *“Como que acá [aborto] está naturalizado, no es un enjuiciamiento que se hace” (Edad 33, Acompañamiento)*. Las sesiones informativas grupales también fueron fundamentales para desdramatizar el aborto. Una participante, que al principio se sentía escéptica acerca de la sesión informativa grupal, relata cómo luego comprendió que era una forma de legitimar el aborto;

*“¿Por qué tengo que ir a una entrevista colectiva? Primero no me gustó un carajo y después yo entendí por qué era colectiva, o por lo menos yo me lo expliqué a través de la experiencia que tuvimos, y eso me gustó… medio como que está bien pensado desde el lugar este de sacarlo de lo oscuro o clandestino o práctica ilegal o a escondidas, el hecho de hacerlo colectivo.” (Edad 36, Clínica)*

Otra participante que fue acompañada por el modelo feminista explicó además cómo las sesiones informativas grupales ayudaron a crear una buena relación amistosa entre las participantes, *“Las chicas lo hacían tipo más ameno, parecía una charla de amigas. Sí, estuvo re bueno. Sí, me sentí muy bien, contenida, estuvo bueno”* *(Edad 30, Acompañamiento)*. El espacio sin críticas que los/las proveedores/as y acompañantes ayudaron a facilitar durante las sesiones informativas grupales, al igual que la escucha empática que las personas que abortaron relatan haber recibido de sus proveedores/as y acompañantes, las ayudó a sentirse escuchadas, validadas y apoyadas y fue un aspecto importante de recibir atención con acompañamiento y contención.

**Opciones para el manejo del dolor**

Las personas entrevistadas informaron haber sentido que los/las proveedores/as y acompañantes tomaron en cuenta sus necesidades y les ofrecieron analgésicos y técnicas no médicas innovadores para el manejo del dolor. Por ejemplo, a las participantes que recibieron atención en la clínica se les dieron varias opciones para el manejo del dolor, incluso bolsas de agua caliente para ponerse sobre el abdomen y la posibilidad de escuchar la música que eligieran durante su AMEU. Una participante describió cómo la música y la conversación con las personas en la habitación ayudó a que se distrajera del dolor;

*““Ellos me pusieron música, Los Redondos, y la verdad es que te soy sincera, yo no sentí ningún dolor…las chicas me hablaban y charlábamos, y estaba con la bolsa de agua caliente como por si sentís dolor.” (Edad 32, Clínica)*

Al reflexionar durante las entrevistas, a menudo enfatizaron estos momentos como representativos de interacciones personales de apoyo durante la atención del aborto. Otra participante que recibió atención en la clínica describió sentirse sorprendida por el esfuerzo que sus proveedores/as hicieron para cubrir sus necesidades individuales de comodidad;

*““Me dijo algo que me sorprendió mucho, me dijo ‘si querés poner música’, que me sorprendió para bien en el sentido de que…a qué nivel están, no sé cómo decirlo, como pensando en la comodidad de que el paciente realmente un momento así lo pase lo mejor posible.” (Edad 21, Clínica)*

Como señala esta participante, para muchas participantes la posibilidad de elegir cómo manejar el dolor las ayudó a sentir que estaban teniendo la mejor experiencia de atención médica posible. En el grupo de acompañamiento feminista, las acompañantes les sugirieron a las participantes que prueben diferentes opciones para manejar el dolor en el hogar, por ejemplo, el uso de compresas calientes, o preparar un espacio en el que tomarían los medicamentos para que se sienta único y cómodo. Por ejemplo, una participante mencionó que vio su show favorito en la televisión y preparó sus refrigerios favoritos, como le había sugerido su acompañante.

*“Ella [la acompañante] me dijo que esté tranquila, que cuanto más tranquila esté iba a ser más rápido y más fácil, me dijo genérate un lindo ambiente, que la persona que esté con vos te transmita cosas buenas, si te gustan los sahumerios prendé un sahumerio, prepárate lo que te guste para comer…, me hice una tarta de dulce de leche, nueces y chocolate…me dijo mirá lo que quieras, lo hice mirando Ru Paul”.* *(Edad 30, Acompañamiento)*

La forma empática y compasiva mediante la cual los/las proveedores/as y acompañantes centraron las necesidades y elecciones de las participantes con respecto a la gestión del dolor ayudó a que sintieran que sus necesidades individuales eran tenidas en cuenta y que estaban en un entorno seguro y cómodo durante sus abortos.

1. **Discusión**

Los resultados de este estudio cualitativo proporcionan un entendimiento de los elementos de la atención interpersonal que fueron más sobresalientes para las personas que se realizan un aborto mediante los modelos de atención clínica y de acompañamiento feminista en Argentina. Los ejemplos específicos de interacciones positivas con proveedores/as y acompañantes ayudan a arrojar luz con respecto a lo que las personas buscan en una buena experiencia de aborto. Las personas entrevistadas resaltaron la comunicación abierta, la provisión de información integral, la atención sin juzgamiento y las opciones para el manejo del dolor como componentes clave de sus experiencias de aborto. Reiteraron de manera consistente cuán imperioso fue y cuánto valoraron la atención interpersonal que se proporcionó con acompañamiento y contención (apoyo emocional continuo, empatía y comprensión).

Estos hallazgos contribuyen a un cuerpo de literatura en aumento que reconoce que la atención interpersonal es una prioridad y documenta el valor de las interacciones con los/las proveedores/as centradas en la persona, empáticas y que brindan apoyo emocional. Nuestros resultados se hacen eco de un estudio reciente con clientes de clínicas de aborto en Kenia que resaltó la importancia de recibir continuamente una atención respetuosa y contenedora durante el proceso del aborto ^19^. Otros estudios concluyeron que, si bien es importante, la atención centrada en la persona a menudo está ausente en los servicios de aborto de todo el mundo^27,28^. Los hallazgos actuales nos alientan a considerar a la calidad de la atención y, más específicamente, a la atención interpersonal como necesaria no solo durante un encuentro único con quien provee o acompaña el aborto, sino desde el momento en que una persona decide realizarse un aborto hasta que se completa el proceso. Esto puede incluir diversos tipos de proveedores/as para una persona, como conversaciones con un/a médico/a de atención primaria o consejeras/os o telefonistas de redes de acompañamiento, personal de recepción, técnicos/as de ultrasonido, farmacéuticos/as que venden las pastillas para el aborto o proveedores/as que ofrecen servicios de seguimiento. Nuestros hallazgos complementan otros estudios que muestran que las personas valoran la comunicación compasiva y contenedora que se proporciona no solo en persona sino también en forma virtual por teléfono, en plataformas en línea o en sistemas de mensajes de texto^29,30^. En entornos legalmente restrictivos, en los que las personas que buscan un aborto podrían no tener acceso a servicios clínicos legales y, en particular, durante las restricciones relacionadas con la pandemia global, estos mecanismos alternativos para la comunicación probablemente se tornen más relevantes y, por lo tanto, se debe supervisar la calidad.

Este estudio resalta diversas formas innovadoras de proporcionar interacciones interpersonales centradas en la persona. Las personas que buscaron sus abortos, tanto en el modelo clínico como el el modelo de acompañamiento feminista, encontraron beneficios en las sesiones informativas grupales previas al aborto. Si bien los estudios han demostrado que la consejería grupal no es suficientemente privada para algunas/os clientes^31^, casi todas/os las/los participantes en este estudio sintieron que las sesiones eran valiosas incluso si habían dudado al principio; escucharon las preguntas que las otras personas tenían, se sintieron menos aisladas/os, y la atmósfera grupal podría haber contribuido a naturalizar las experiencias de aborto entre las/los participantes. Además de las sesiones informativas grupales, La Revuelta y FUSA proporcionaron muchas oportunidades para que las/los participantes realicen preguntas y reciban apoyo de los/las proveedores/as en forma personalizada. Además, algunas/os participantes informaron sentirse impresionadas/os por los intentos que realizaron los/las proveedores/as y acompañantes para hacer que la experiencia del aborto no fuera solo cómoda sino también placentera. Esto incluyó poner música durante los abortos en la clínica, ofrecer bolsas de agua caliente durante y después del procedimiento, y proporcionar sugerencias sobre cómo preparar sus espacios en el hogar para facilitar la experiencia de aborto autogestionado. Estos enfoques innovadores se centraron en las necesidades de la/del cliente y la/lo ayudaron a sentirse cuidada/o y apoyada/o durante su aborto. Las estrategias que ayudan a naturalizar el aborto y les ofrecen opciones y autonomía a las personas que buscan un aborto son particularmente dignas de atención dentro de la atención del aborto, ya que ayudan a las personas a sentirse empoderadas en sus decisiones y podrían ayudar a derribar algunos de los estigmas internalizados o estigmas sociales que rodean al aborto.

Este estudio agrega matices a dominios que han emergido en los marcos de atención centrados en la persona que se encuentran en la literatura. Los detalles que se ofrecen en estas narrativas tienen implicancias para cómo desarrollamos o adaptamos herramientas para medir la calidad de la atención desde la perspectiva de la/del cliente. Por ejemplo, además de recibir información integral acerca del proceso de aborto, las/los participantes sintieron que fue importante que la información fuera proporcionada en una manera que las/los ayudara a sentirse escuchadas/os, reconfortadas/os, validadas/os y libres de realizar preguntas aclaratorias. También mencionaron que valoraban haber podido decidir cómo manejar el dolor durante su aborto en la clínica durante un aborto quirúrgico y en el hogar en los abortos con medicamentos. Si los indicadores abordan solamente si una persona recibió información o manejo del dolor, podrían no evaluar con éxito *cómo* recibió ese elemento de la atención. Por otro lado, si los indicadores solo le preguntan a una persona si sintió que recibió atención compasiva y respetuosa, podrían no capturar con éxito en qué punto durante el proceso la persona se sintió, o no se sintió, respetada. Sudhinaraset et al. recientemente desarrollaron y validaron una escala de atención del aborto centrada en la persona con base en datos de clientes de aborto en Kenia que incluyó subescalas para medir la atención respetuosa y contenedora, y también la comunicación y la autonomía^32^. Estos dominios se reflejan en nuestros datos, lo que sugiere que los aspectos claves de la atención centrada en la persona pueden trasladarse a contextos políticos y sociales. Por ejemplo, las descripciones detalladas de atención de alta calidad de este estudio en Argentina pueden contribuir a investigaciones en el futuro que exploren cómo se pueden adoptar esta escala o mediciones en el futuro al contexto argentino. Esto es particularmente importante dado que el gobierno se prepara para expandir el acceso al aborto legal en establecimientos públicos en todo el país. Será fundamental que, durante el desarrollo de las directrices de atención, en la capacitación de los/las proveedores/as y en la supervisión y mejora de los servicios se incorporen los valores y preferencias de las personas que obtienen un aborto.

Existen varias limitaciones que se deben considerar al interpretar los hallazgos de este estudio. Primero, este estudio convocó a participantes de dos centros conocidos por proporcionar atención del aborto de alta calidad y, por lo tanto, no representan las experiencias en todo un rango de servicios. Si bien se necesita más investigación para comprender las experiencias de las personas en establecimientos públicos, los hallazgos actuales ofrecen un entendimiento único de las preferencias y prioridades en la atención interpersonal. Segundo, no le preguntamos a las personas acerca de su identidad de género, y, por lo tanto, no podemos decir nada acerca del rango de géneros presente dentro de este documento ni acerca de cómo la identidad de género podría haber afectado las experiencias de atención del aborto de las personas. Tercero, las sedes de ambos centros de reclutamiento estaban en áreas urbanas, por lo tanto, las perspectivas no representan a las áreas rurales de Argentina. Por último, los/las proveedores/as y acompañantes en los dos centros fueron responsables de invitar a las personas a participar en el estudio, lo que podría haber influido en quién decidió participar y podría haber limitado las opiniones negativas que se compartieron en la entrevista.

1. **Conclusión**

Este estudio identificó los elementos de la atención interpersonal que fueron más sobresalientes para las personas que buscan un aborto en clínicas y con grupos de acompañamiento en Argentina. Nuestros hallazgos sugieren que el hecho de recibir una atención compasiva y contenedora durante el aborto fue, de manera consistente, uno de los aspectos clave de la atención. Si bien los contextos legales y culturales pueden modificar lo que las personas que buscan un aborto consideran que es más importante, planteamos que la sensación de confianza, empatía y respeto de los/las proveedores/as que emergió de este estudio se puede aplicar a todos los entornos y modelos de atención. Dado el cambio global que existe hacia el aborto con medicamentos y la reciente expansión de la telemedicina durante la pandemia global de Covid-19, este estudio nos recuerda que las interacciones interpersonales tienen mucha importancia para la experiencia general de calidad de la atención que tiene una persona, incluso cuando se provee apoyo para un aborto de manera virtual. Además, dado este momento crítico en la provisión del aborto en Argentina, esperamos que estos hallazgos informen a la provisión y evaluación de la atención del aborto centrada en la persona en el país.

1. **Lista de abreviaturas**

Casa FUSA, Centro para Atención de Adolescentes y Jóvenes (FUSA): Una clínica de atención reproductiva integral en Buenos Aires

Ibis Reproductive Health (Ibis)

Colectiva Feminista La Revuelta (La Revuelta)

Aspiración manual (AVM)

Aborto con medicamentos (AM)

1. **Referencias**

1. Ley N^o^ 27.610 - Acceso a la Interrupción Voluntaria del Embarazo (IVE), obligatoriedad de brindar cobertura integral y gratuita | Argentina.gob.ar. Accessed January 25, 2022. https://www.argentina.gob.ar/noticias/ley-no-27610-acceso-la-interrupcion-voluntaria-del-embarazo-ive-obligatoriedad-de-brindar

2. Szulik D, Zamberlin N. La legalidad oculta: Percepciones de estigma en los recorridos de mujeres que descubren y acceden a la interrupción legal del embarazo por causal salud. *Sex Salud y Soc (Rio Janeiro)*. 2020;(34):46-67. doi:10.1590/1984-6487.SESS.2020.34.04.A

3. Serna Botero S, Cárdenas R, Zamberlin N. ¿De qué está hecha la objeción? Relatos de objetores de conciencia a servicios de aborto legal en Argentina, Uruguay y Colombia. *Sex Salud y Soc (Rio Janeiro)*. 2020;(33):137-157. doi:10.1590/1984-6487.SESS.2019.33.08.A

4. Gomperts R, Jelinska K, Davies S, Gemzell-Danielsson K, Kleiverda G. Using telemedicine for termination of pregnancy with mifepristone and misoprostol in settings where there is no access to safe services. *BJOG An Int J Obstet Gynaecol*. 2008;115(9):1171-1178. doi:10.1111/j.1471-0528.2008.01787.x

5. Dzuba IG, Winikoff B, Peña M. Medical abortion: A path to safe, high-quality abortion care in Latin America and the Caribbean. *Eur J Contracept Reprod Heal Care*. 2013;18(6):441-450. doi:10.3109/13625187.2013.824564

6. Darney BG, Kapp N, Andersen K, et al. Definitions, measurement and indicator selection for quality of care in abortion. *Contraception*. 2019;100(5):354-359. doi:10.1016/j.contraception.2019.07.006

7. Dennis A, Blanchard K, Bessenaar T. Identifying indicators for quality abortion care: A systematic literature review. *J Fam Plan Reprod Heal Care*. 2017;43(1):7-15. doi:10.1136/jfprhc-2015-101427

8. Donnelly KZ, Dehlendorf C, Reed R, Agusti D, Thompson R. Adapting the Interpersonal Quality in Family Planning care scale to assess patient perspectives on abortion care. *J Patient-Reported Outcomes*. 2019;3(1):3. doi:10.1186/s41687-018-0089-7

9. Dennis A, Blanchard K, Bessenaar T. Identifying indicators for quality abortion care: A systematic literature review. *J Fam Plan Reprod Heal Care*. 2017;43(1):7-15. doi:10.1136/jfprhc-2015-101427

10. WHO | What are integrated people-centred health services? *WHO*. Published online 2018. Accessed February 22, 2021. http://www.who.int/servicedeliverysafety/areas/people-centred-care/ipchs-what/en/

11. Holt K, Zavala I, Quintero X, et al. Women’s preferences for contraceptive counseling in Mexico: Results from a focus group study. *Reprod Health*. 2018;15(1). doi:10.1186/s12978-018-0569-5

12. Renfrew MJ, McFadden A, Bastos MH, et al. Midwifery and quality care: Findings from a new evidence-informed framework for maternal and newborn care. *Lancet*. 2014;384(9948):1129-1145. doi:10.1016/S0140-6736(14)60789-3

13. Jain AK, Hardee K. Revising the FP Quality of Care Framework in the Context of Rights-based Family Planning. *Stud Fam Plann*. 2018;49(2):171-179. doi:10.1111/sifp.12052

14. Holt K, Dehlendorf C, Langer A. Defining quality in contraceptive counseling to improve measurement of individuals’ experiences and enable service delivery improvement. *Contraception*. 2017;96(3):133-137. doi:10.1016/j.contraception.2017.06.005

15. Dehlendorf C, Henderson JT, Vittinghoff E, et al. Association of the quality of interpersonal care during family planning counseling with contraceptive use. *Am J Obstet Gynecol*. 2016;215(1):78.e1-78.e9. doi:10.1016/j.ajog.2016.01.173

16. Abdel-Tawab N, Roter D. The relevance of client-centered communication to family planning settings in developing countries: Lessons from the Egyptian experience. *Soc Sci Med*. 2002;54(9):1357-1368. doi:10.1016/S0277-9536(01)00101-0

17. Holt K, Caglia JM, Peca E, Sherry JM, Langer A. A call for collaboration on respectful, person-centered health care in family planning and maternal health. *Reprod Health*. 2017;14(1):1-3. doi:10.1186/s12978-017-0280-y

18. Taylor D, Postlethwaite D, Desai S, et al. Multiple Determinants of the Abortion Care Experience: From the Patient’s Perspective. *Am J Med Qual*. 2013;28(6):510-518. doi:10.1177/1062860613484295

19. Cotter SY, Sudhinaraset M, Phillips B, et al. Person-centred care for abortion services in private facilities to improve women’s experiences in Kenya. *Cult Health Sex*. 2021;23(2):224-239. doi:10.1080/13691058.2019.1701083

20. Regmi K, Madison J. Ensuring patient satisfaction with second-trimester abortion in resource-poor settings. *Int J Gynecol Obstet*. 2010;108(1):44-47. doi:10.1016/j.ijgo.2009.08.005

21. Becker D, Díaz-Olavarrieta C, Juárez C, García SG, Sanhueza P, Harper CC. Clients’ perceptions of the quality of care in Mexico City’s public-sector legal abortion program. *Int Perspect Sex Reprod Health*. 2011;37(4):191-201. doi:10.1363/3719111

22. Ponce de León RG, Billings DL, Barrionuevo K. Woman-centered post-abortion care in public hospitals in Tucumán, Argentina: Assessing quality of care and its link to human rights. *Health Hum Rights*. 2006;9(1):175-201. doi:10.2307/4065395

23. Jacobson LE, Ramirez AM, Bercu C, Katz A, Gerdts C, Baum SE. Understanding the Abortion Experiences of Young People to Inform Quality Care in Argentina, Bangladesh, Ethiopia, and Nigeria: *https://doi.org/101177/0044118X211011015*. Published online April 24, 2021. doi:10.1177/0044118X211011015

24. *Medical Management of Abortion*.; 2018. Accessed January 22, 2021. http://apps.who.int/

25. Kruk ME, Kelley E, Syed SB, Tarp F, Addison T, Akachi Y. Measuring quality of health-care services: What is known and where are the gaps? *Bull World Health Organ*. 2017;95(6):390-390A. doi:10.2471/BLT.17.195099

26. Sudhinaraset M, Afulani P, Diamond-Smith N, Bhattacharyya S, Donnay F, Montagu D. Advancing a conceptual model to improve maternal health quality: The person-centered care framework for reproductive health equity. *Gates Open Res*. 2017;1. doi:10.12688/gatesopenres.12756.1

27. Altshuler AL, Whaley NS. The patient perspective: perceptions of the quality of the abortion experience. *Curr Opin Obstet Gynecol*. 2018;30(6):407-413. doi:10.1097/GCO.0000000000000492

28. Gerdts C, Raifman S, Daskilewicz K, Momberg M, Roberts S, Harries J. Women’s experiences seeking informal sector abortion services in Cape Town, South Africa: A descriptive study. *BMC Womens Health*. 2017;17(1):1-10. doi:10.1186/S12905-017-0443-6/TABLES/5

29. Baum SE, Ramirez AM, Larrea S, et al. “It’s not a seven-headed beast”: abortion experience among women that received support from helplines for medication abortion in restrictive settings. *Health Care Women Int*. 2020;41(10):1128-1146. doi:10.1080/07399332.2020.1823981

30. Fix L, Seymour JW, Sandhu MV, Melville C, Mazza D, Thompson TA. At-home telemedicine for medical abortion in Australia: A qualitative study of patient experiences and recommendations. *BMJ Sex Reprod Heal*. 2020;46(3):172-176. doi:10.1136/bmjsrh-2020-200612

31. Birdsey G, Crankshaw TL, Mould S, Ramklass SS. Unmet counselling need amongst women accessing an induced abortion service in KwaZulu-Natal, South Africa. *Contraception*. 2016;94(5):473-477. doi:10.1016/J.CONTRACEPTION.2016.07.002

32. Sudhinaraset M, Landrian A, Afulani PA, Phillips B, Diamond-Smith N, Cotter S. Development and validation of a person-centered abortion scale: The experiences of care in private facilities in Kenya. *BMC Womens Health*. 2020;20(1). doi:10.1186/s12905-020-01071-w

**Archivo complementario I: Guía para la entrevista (en español)**

***[Nota a entrevistadora: las instrucciones van a estar en negrita para que sean fáciles de leer; no debieran ser dichas en voz alta]***

Hola, mi nombre es ____________. Estoy conduciendo esta entrevista de parte de ***[organización]*** e Ibis Reproductive Health. Muchas gracias por acceder a participar. Quiero resaltar que para asegurarse de que las mujeres reciban la mejor calidad de servicios, te estamos pidiendo que compartas tus pensamientos honestos sobre los servicios que recibiste. Nada de lo que digas hoy va a lastimar mis sentimientos, y nada de lo que digas te va a ser atribuido, y no va a afectar tu habilidad para acceder a servicios en el futuro.

Como mencioné en el proceso de consentimiento, hoy me gustaría explorar tus pensamientos, opiniones, y experiencias con servicios de salud sexual y reproductiva, incluyendo aborto. Lo que compartas nos ayudará a mejorar la calidad de estos servicios. No estoy buscando un tipo de respuesta en particular, solo tu opinión honesta.

Para proteger tu confidencialidad y privacidad, tu nombre nunca será usado en conexión a la información que compartas. Por favor recordá que tu participación es puramente voluntaria, no necesitás responder ninguna pregunta que te incomode, y podés terminar la entrevista en cualquier momento sin ningún tipo de penalización.

Vamos a empezar. Voy a empezar a grabar ahora.

***[Empezar grabación]***

***[Decir el número de identificación, nombre de la entrevistadora y la fecha al comienzo de la grabación]***

**Sección 1. Introducción**

Me gustaría empezar preguntándote un poquito sobre vos.

1. ¿Me podrías contar un poco sobre vos? *[Por ejemplo edad, trabajo, familia, etc.]*

**Sección 2. Definiendo cuidado de alta calidad**

Ahora voy a hacer una transición para preguntarte sobre servicios de salud. Por favor pensá sobre cuando vos o tu familia han estado enfermos y necesitaron buscar cuidado médico.

1. ¿En dónde buscan servicios de cuidado médico vos o tu familia usualmente?
   1. ¿Por qué van a buscar servicios de cuidado médico allá?

A veces recibimos servicios de salud que son buenos, a veces muy malos, y a veces en el medio.

1. Por favor describí un momento reciente en el que hayas recibido cuidados de salud muy buenos de un doctor/a, enfermero/a o algún otro proveedor de servicios o cuidados de salud.
2. ¿Qué hizo que el servicio sea muy bueno?
3. Ahora por favor describí un momento reciente en el que vos o tu familia hayan recibido cuidados de salud inaceptables o malos de un proveedor de salud.
   1. ¿Qué hizo que el cuidado sea malo?
4. En tu opinión, ¿qué es lo que hace que algunos servicios de salud sean buenos o sean malos?
5. ¿A dónde van las mujeres en tu comunidad para obtener cuidado prenatal?
   1. ¿Y para métodos anticonceptivos?
   2. ¿Cómo saben las mujeres en tu comunidad a dónde ir para obtener buenos cuidados para estos servicios?

**Sección 3. Antes del aborto**

Ahora me gustaría preguntarte algunas preguntas sobre tu embarazo reciente.

1. ¿En general, cómo te sentías o cuáles eran tus creencias respecto al aborto en general antes de decidir tener un aborto?
2. ¿Qué habías escuchado sobre el aborto antes de obtener servicios de aborto?
   1. ¿Sobre la seguridad o riesgos potenciales del aborto?
   2. ¿Sobre los riesgos o consecuencias potenciales después de un aborto?
   3. ¿Sobre cómo serías tratada?
   4. ¿Sobre las leyes sobre cuando una mujer puede obtener un aborto?
3. ¿Dónde aprendiste esta información?
4. ¿Cómo decidiste buscar servicios de aborto?
5. ¿Hiciste algo para intentar terminar con ese embarazo antes de contactar a La Revuelta/FUSA? Contame sobre eso.
6. ¿Cómo te enteraste de La Revuelta/FUSA?
   1. ¿Qué habías escuchado sobre La Revuelta/FUSA?
7. ¿Qué te hizo decidir buscar servicios de La Revuelta/FUSA?
   1. ¿Escuchaste historias sobre otras mujeres obteniendo servicios de aborto que influenciaron tu decisión sobre dónde ir? Contame sobre eso.
8. ¿Qué expectativas tenías sobre cómo serías tratada al recibir cuidados de aborto?
   1. ¿Y tenías algún miedo? ¿Cuál era tu mayor miedo?
9. ¿Hablaste con un/a proveedor/a de salud sobre tu embarazo antes de contactar a La Revuelta/FUSA por primera vez?

***[SI ES ASI]***

1. Contame sobre eso.
2. ¿Qué te dijeron?
3. ¿Cómo te trataron?
4. ¿Más o menos cual era tu edad gestacional cuando contactaste a La Revuelta/FUSA? Sentite libre de hacer una estimación si no te acordás exactamente.

**Sección 4. Durante el aborto**

Tengo curiosidad de escuchar más sobre tu experiencia con la telefonista/acompañante

1. ¿Cómo fuiste tratada durante tus interacciones con La Revuelta/FUSA?
   1. ¿Qué te hizo sentir de esa manera?
2. ¿Hay algo que dijeron que te asustó? ¿Por qué?
3. ¿De qué formas te hicieron sentir preparada o no preparada para el aborto?
4. ¿Qué tipo de información recibiste de parte de la telefonista/acompañante que haya sido difícil de entender, si es que esto ocurrió?
5. ¿Qué hizo la telefonista/acompañante para proteger o no tu privacidad?
   1. ¿Qué tan importante fue esto para vos?
6. ¿En algún punto te sentiste juzgada durante tu experiencia con La Revuelta/FUSA? Contame sobre eso
7. ¿Tuviste alguna otra interacción negativa con otras telefonistas/acompañantes? Contame sobre eso.
8. Contame sobre la información que recibiste sobre anticonceptivos que recibiste después de tu aborto.
   1. ¿Había alguna información que querías pero que no recibiste en relación a métodos anticonceptivos? Contame sobre eso.
9. ¿Quién estaba con vos cuando tomaste las pastillas, si es que había alguien? ¿De qué formas te apoyó o no esta persona?
10. ¿Cuántas veces hablaste con la telefonista/acompañante durante todo el proceso?

**Sección 5. Después del aborto**

1. ¿Fuiste a ver a un doctor/a o fuiste a una clínica después del aborto/terminar el embarazo? ¿Por qué si? ¿Por qué no?

***[SI ES ASI]***

1. ¿Qué le dijiste al/a la proveedor/a sobre lo que pasó?
2. ¿Qué tipo de tratamiento médico recibiste?
3. ¿Cómo te trataron?
4. ¿Cuál fue la mejor parte del cuidado que recibiste? ¿Por qué?
5. ¿Cuál fue la peor parte del cuidado que recibiste? ¿Por qué?
6. Pensando sobre el costo del aborto, ¿cómo lo describirías? ¿Qué tan manejable fue para vos?
7. ¿Creés que tu edad afectó la manera que fuiste tratada?
   1. ¿Creés que la experiencia hubiese sido igual o distinta para una amiga tuya que es mucho más grande que vos? ¿Más chica? ¿Por qué?
8. ¿Creés que tu estado civil afectó cómo fuiste tratada?
9. ¿Creés que la experiencia hubiese sido igual o distinta para una amiga tuya que está **(*casada or no casada)***  ***[Elegir la experiencia opuesta de la participante]***

**Sección 6. Reflexiones sobre la experiencia del aborto**

1. Si alguien nunca hubiese escuchado de La Revuelta/FUSA, ¿cómo las describirías a esta persona?
2. ¿Qué hubiese hecho la experiencia con La Revuelta/FUSA mejor?
3. ¿Cómo se compara, o no, tu experiencia recibiendo servicios de aborto con respecto a tus expectativas?
4. ¿Habías tenido una experiencia de aborto antes de este embarazo reciente?
5. ¿Y cómo se compara esta experiencia con tu aborto previo?

***[Por ejemplo: seguridad, dolor, prontitud y experiencia con proveedor/***

1. ¿Qué consejo le darías a una amiga que también está buscando cómo terminar su embarazo? ¿Qué debería saber?
2. Si tuvieras que describir las tres partes más importantes del mejor cuidado de aborto, ¿qué tres partes dirías? Por favor, mencioná las partes que sientas que son las más importantes para vos, sin importar que tan grandes o chicas.

Por último….

1. ¿Cómo fue participar en esta entrevista?
2. Antes de terminar, me gustaría preguntarte algunas preguntas más sobre vos. Le preguntamos esto a todas las personas con las que hablamos.
3. ¿Cuántos años tenés? _________
4. ¿Cuál es tu estado sentimental en este momento?
5. ¿Cuántos hijos tenés, si es que tenés hijos? ____________
6. ¿Estás trabajando en este momento? ¿Estudiando? ¿Las dos?

**-- Gracias por tomarte el tiempo de hacer esta entrevista con nosotras--**

1. Cuando es posible, usamos “persona embarazada” y “personas” en este documento para reconocer la diversidad de personas con útero y la diversidad de experiencias de embarazo y aborto. Especificamos “mujeres” cuando las/los autoras/es de otras publicaciones se refieren a las personas como mujeres. Reconocemos que, en la literatura sobre embarazo y aborto, las experiencias de individuos intersexuales, transexuales y no binarios tienen poca representación, y que este análisis no fue estructurado para abordar esta brecha. [↑](#footnote-ref-1)
2. En este manuscrito usamos el término “personas” ya que no le preguntamos a las/los participantes acerca de su identidad de género. [↑](#footnote-ref-2)
